# PDHA1 Hyperactivation Orchestrates Metabolic Reprogramming Promoting Endothelial Senescence

**DOI:** 10.34133/research.1398

**Published:** 2026-08-20

**Authors:** Wen-Jing Zhong, Nan-Shi-Yu Yang, Chen-Yu Zhang, Yu-Biao Liu, Ling Jin, An-Jun Ou, Hui Chen, Juan Li, Yong Zhou, Bai-Mei He, Jia-Xi Duan

**Affiliations:** ^1^ Department of Geriatric Respiratory and Critical Care Medicine, Xiangya Hospital, Central South University, Changsha, Hunan 410008, China.; ^2^ Department of Physiology, Xiangya School of Basic Medical Sciences, Central South University, Changsha, Hunan 410078, China.; ^3^ National Experimental Teaching Demonstration Center for Medical Function, Central South University, Changsha, Hunan 410078, China.; ^4^ National Clinical Research Center for Geriatric Disorders, Xiangya Hospital, Central South University, Changsha, Hunan 410008, China.

## Abstract

While recent studies have established links between metabolic reprogramming and inflammatory senescence, the specific metabolic drivers in vascular aging remain incompletely defined. Here, we systematically characterized senescent phenotypes and targeted metabolomic profiles in primary aging endothelial cells, identifying a pyruvate dehydrogenase E1 component subunit alpha (PDHA1)-dependent metabolic shift as a hallmark of cellular senescence. Using a D-galactose-induced senescence model, we demonstrated that endothelial-specific *Pdha1* knockdown alleviated pulmonary vascular endothelial senescence and associated functional decline. Further investigation revealed that PDHA1 hyperactivation disrupts mitochondrial homeostasis, leading to excessive mitochondrial reactive oxygen species production, oxidative mitochondrial DNA damage, and subsequent cytosolic mitochondrial DNA release, thereby triggering cyclic GMP-AMP synthase-mediated senescence. Mechanistically, decreased lactylation of PDHA1 at lysine 336 potentiated its activity and promoted dephosphorylation at serine 293. This posttranslational cross talk enhanced PDHA1 activation and drove a prosenescent metabolic shift. Together, our results elucidate that a previously unrecognized PDHA1 hyperactivation promotes endothelial senescence.

## Introduction

Age-related vascular dysfunction is a key contributor to the pathogenesis of numerous pulmonary disorders, including chronic obstructive pulmonary disease (COPD) and pulmonary fibrosis (PF) [1]. An aging lung exhibits characteristic declines in function, impaired repair capacity, structural remodeling, and increased susceptibility to injury and disease [2]. Growing evidence supports the vascular theory of aging, which positions vascular senescence as a primary driver of systemic aging processes, ultimately leading to progressive lung function deterioration and elevated risk of respiratory conditions in older people [3]. Pulmonary vascular endothelial cells (PVECs), the principal structural component of the alveolar–capillary barrier, perform essential physiological functions [4]. During senescence, PVECs undergo profound alterations in gene expression, proliferative capacity, and cellular architecture. These changes undermine endothelial integrity by compromising regenerative potential, angiogenic responses, and vascular reactivity, thereby accelerating disease processes and promoting age-related vascular pathologies [5–7]. Among the multiple factors known to induce endothelial senescence, dysregulation of glucose metabolism pathways emerges as a particularly important contributor. However, the precise mechanisms through which metabolic reprogramming drives vascular aging remain incompletely elucidated.

Recent studies have positioned glucose metabolic reprogramming as a pivotal regulator of the aging process. Initially identified in cancer cells as the Warburg effect, this metabolic shift is characterized by enhanced aerobic glycolysis and suppressed oxidative phosphorylation (OXPHOS) [8]. In immune cells, metabolic dysregulation promotes chronic inflammation and cellular senescence [9], a mechanism exemplified by our previous finding that macrophage glycolytic reprogramming drives inflammation [10]. Consistently, senescent macrophages or T cells up-regulate aerobic glycolysis to maintain their characteristic cell cycle arrest while preserving metabolic activity [11,12]. Notably, the metabolic profile of endothelial cells (ECs) differs fundamentally from that of most immune cell types [13,14]. Proliferating ECs exhibit a distinct metabolic phenotype, predominantly relying on glycolysis for energy production, generating approximately 85% of their adenosine triphosphate (ATP) through this pathway [13]. Recent studies have established a clear link between metabolic reprogramming and EC senescence. For instance, glycolysis-supplied serine biosynthesis safeguards ECs from senescence by coupling phosphoglycerate dehydrogenase (PHGDH) to pyruvate kinase M2 (PKM2) stability and nuclear transcriptional regulation [15]. Similarly, hypoxia remodels the lactate dehydrogenase isozyme profile, boosts glycolytic flux and nicotinamide adenine dinucleotide (NAD^+^) recycling, and activates BNIP3-mediated mitophagy, thereby suppressing senescence in endothelial progenitor cells [16]. Despite these advances, the precise alterations in specific glycolytic enzymes and intermediate metabolites during endothelial senescence, and how they functionally contribute to the senescent phenotype beyond known pathways, remain incompletely characterized.

The pyruvate dehydrogenase complex (PDHC) occupies a central position in cellular metabolism, serving as the critical link between glycolysis and mitochondrial OXPHOS by catalyzing the irreversible conversion of pyruvate to acetyl-CoA [17]. Located within the mitochondrial matrix, this multienzyme complex is organized around 3 core components: the rate-limiting E1 enzyme (pyruvate dehydrogenase [PDH], composed of catalytic PDHA and regulatory PDHB subunits), E2 (dihydrolipoyl transacetylase), and E3 (dihydrolipoyl dehydrogenase) [18]. The catalytic activity of pyruvate dehydrogenase E1 component subunit alpha (PDHA1), which harbors the primary active site, is precisely regulated through reversible phosphorylation. Phosphorylation at serine 293 (S293) represents a key inhibitory mechanism that suppresses PDHC function [19]. Under hypoxic conditions, phosphorylation of PDHA S293 inhibits PDHC activity, thereby promoting glycolysis and oncogenic behavior in lung cancer [20]. Additionally, the inactivation of PDHA1 through S293 phosphorylation not only diminishes enzymatic activity but also disrupts subsequent activity-dependent phosphorylation events [21]. This mechanism extends to chemotherapy resistance, where PDHA1 inactivation facilitates metabolic reprogramming, thereby contributing to the adaptive metabolic shift in cancer cells [22]. Despite extensive characterization of PDHA1 regulation in cancer metabolism, its potential role in endothelial senescence remains largely unexplored.

Our study revealed that attenuated glycolysis coupled with enhanced, PDHA1-driven OXPHOS constituted a critical mechanism underlying pulmonary EC senescence. We demonstrated that this elevated OXPHOS induced mitochondrial dysfunction, which subsequently activated the cyclic GMP-AMP synthase (cGAS)–stimulator of interferon genes (STING) pathway and instigated senescence-associated signaling. Importantly, endothelial-specific *Pdha1* depletion through gene therapy alleviated age-related pulmonary vascular senescence and associated functional deficits. Mechanistically, the enhanced activity of PDHA1 was attributed to reduced lactylation at K336, which facilitated S293 dephosphorylation. Collectively, our findings provide novel insights into the metabolic regulation of endothelial senescence.

## Results

### Aged PVECs exhibit divergent glucose metabolic reprogramming, favoring OXPHOS over glycolysis

**Fig. 1.**
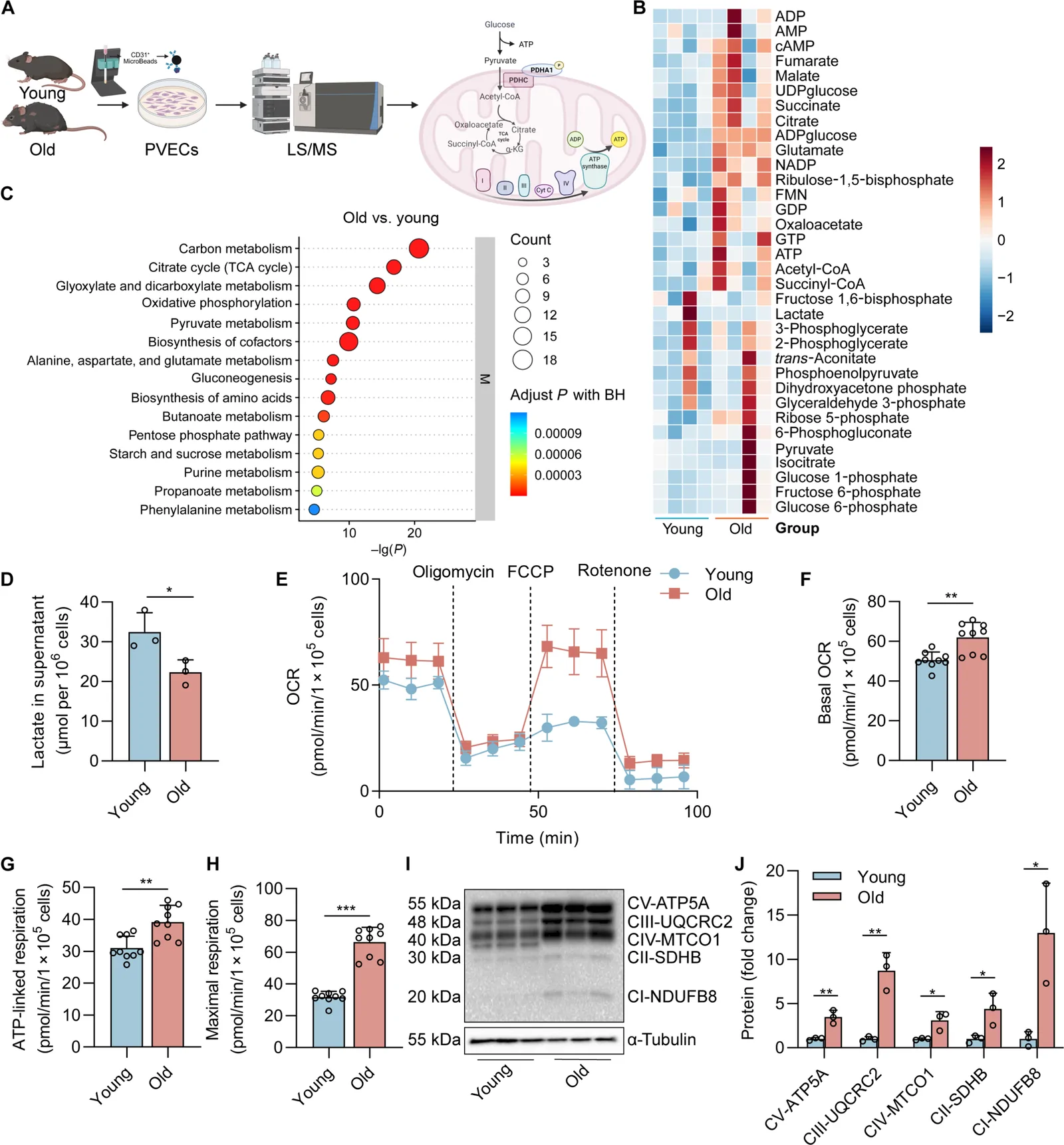
Aged pulmonary vascular endothelial cells (PVECs) instigate a nonclassical glucose metabolic reprogramming characterized by enhanced oxidative phosphorylation (OXPHOS) in mice. (A) Schematic diagram of the metabolomics analysis workflow for mouse primary PVECs from aged (18-month-old) mice. (B) Heatmap showing targeted metabolomic profiles in the mouse primary PVECs isolated from aged mice. (C) Targeted metabolomics analysis depicting top metabolomic pathways enriched in aging PVECs. *n* = 4. (D) Analysis of lactate production in aging PVECs. *n* = 3. (E and F) The oxygen consumption rate (OCR) was obtained using Seahorse XF24 Analyzer. (G and H) Statistical analyses of adenosine triphosphate (ATP)-linked respiration and maximal respiration in aged mouse primary PVECs. (I and J) OXPHOS-related proteins: ATP5A, UQCRC2, MTCO1, SDHB, and NDUFB8 in PVECs from aged and control mice were detected by Western blot. *n* = 3. *P < 0.05, **P < 0.01, and ***P < 0.001.

To assess metabolic alterations during EC senescence, we first isolated PVECs from aged mice (Fig. S1A and B). Their senescent phenotype was confirmed through multiple established markers: positive senescence-associated β-galactosidase (SA-β-gal) staining (Fig. S1C and D), marked up-regulation of senescence-associated messenger RNAs (mRNAs; *p16*, *p19*, *p21*, *p53*, *Il-1β*, and *Il-18*; Fig. S1E and F), and increased protein levels of p53, p21, p16, and phosphorylated histone H2AX (γ-H2AX) (Fig. S1G and H). Targeted metabolomic profiling of these senescent PVECs identified different abundant intermediate metabolites compared to young controls (Fig. 1A and B). Notably, we observed an accumulation of tricarboxylic acid (TCA) cycle intermediates alongside a reduction in glycolytic metabolites (Fig. 1B). This metabolic rewiring was further supported by Kyoto Encyclopedia of Genes and Genomes (KEGG) pathway analysis, which highlighted perturbations in OXPHOS and glucose metabolism (Fig. 1C). Metabolically, aged PVECs exhibited suppressed glycolytic flux, as evidenced by reduced lactate production (Fig. 1D). In contrast, OXPHOS was markedly enhanced, demonstrated by an elevated oxygen consumption rate (OCR; Fig. 1E and F), ATP-linked respiration (Fig. 1G), and maximal respiration (Fig. 1H). Consistent with these bioenergetic changes, Western blot analysis confirmed the up-regulation of key OXPHOS components, including ATP5A, UQCRC2, MTCO1, SDHB, and NDUFB8 (Fig. 1I and J). To determine whether this metabolic shift is a general feature of EC senescence, we employed a D-galactose (D-gal)-induced senescence model in both mouse primary PVECs and human umbilical vein endothelial cells (HUVECs). D-gal-induced senescent cells recapitulated the metabolic phenotype observed in naturally aged PVECs, exhibiting diminished glycolysis and enhanced OXPHOS (Fig. S2). These results indicate that senescent ECs undergo a metabolic shift characterized by enhanced OXPHOS and limited glycolytic activity.

### PDHA1 hyperactivation in senescent pulmonary ECs

**Fig. 2.**
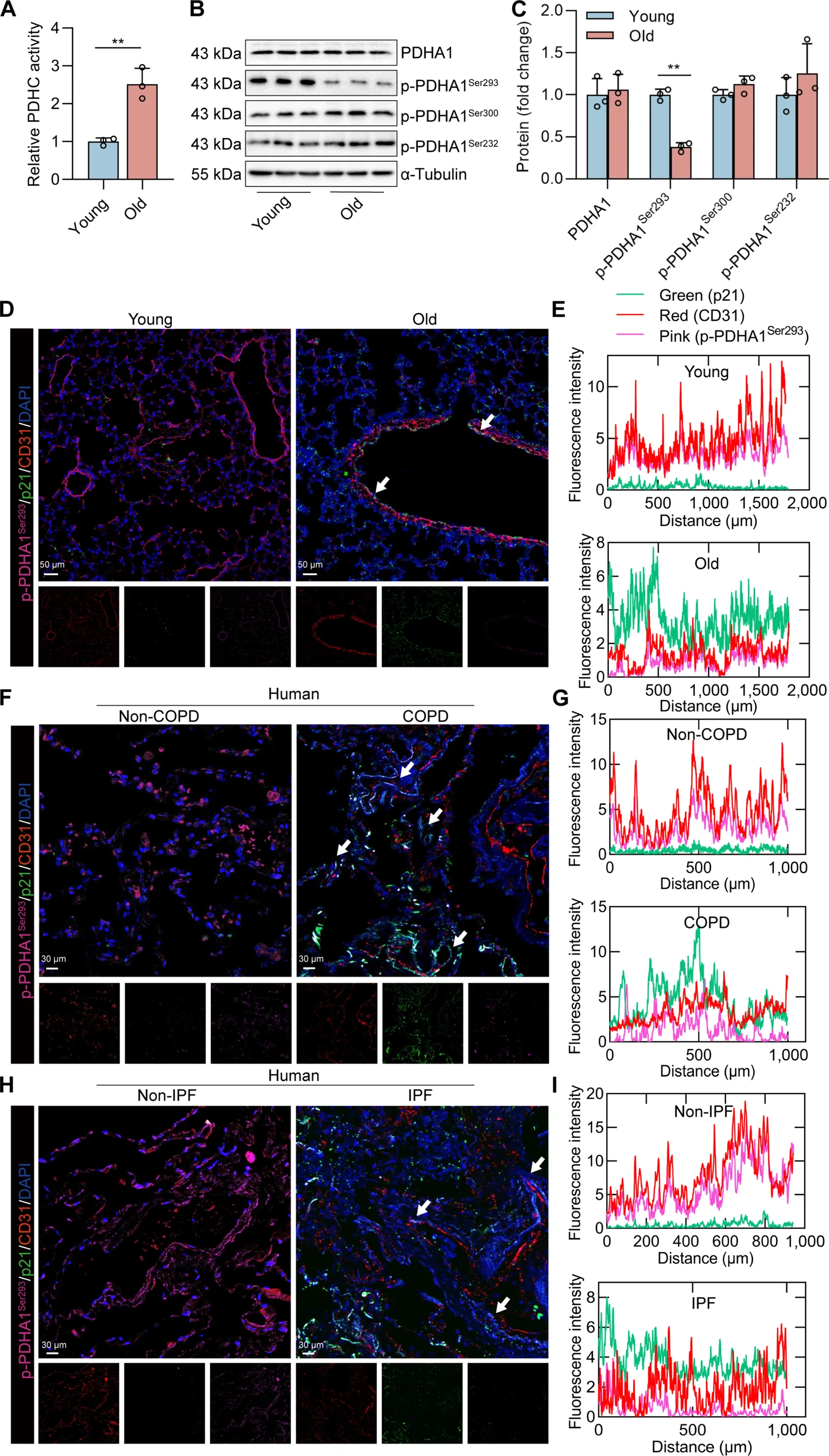
Enhanced pyruvate dehydrogenase E1 component subunit alpha (PDHA1) activity in senescent pulmonary endothelial cells. (A) Relative pyruvate dehydrogenase complex (PDHC) activity in mouse primary pulmonary vascular endothelial cells (PVECs) isolated from aged mice. (B and C) The phosphorylation level at PDHA1 S293, S300, and S232 was determined in PVECs isolated from young and aged mice. *n* = 3. (D) The colocalization of p-PDHA1^S293^, p21, and CD31 was assessed by immunofluorescence staining (IF) in lung tissues obtained from both young and aged mice. Scale bar = 50 μm. (E) The fluorescence intensity curve shows the distribution of PDHA1^S293^ (pink), p21 (green), and CD31 (red) along the cells. (F and G) p-PDHA1^S293^, p21, and CD31 colocalization was determined by IF in lung tissue from non-chronic obstructive pulmonary disease (non-COPD) donors and COPD patients. Scale bar = 30 μm. (H and I) The colocalization of p-PDHA1^S293^, p21, and CD31 was assessed by immunofluorescence in lung tissues obtained from non-idiopathic pulmonary fibrosis (non-IPF) donors and IPF patients. Scale bar = 30 μm. **P < 0.01.

Pyruvate occupies a central metabolic position, bridging glycolysis and the TCA cycle. Its fate is primarily determined by the competing activities of lactate dehydrogenase A, which reduces pyruvate to lactate to sustain glycolytic flux, and PDHC, which directs carbon into the TCA cycle via oxidative decarboxylation to acetyl-CoA [23]. Among the regulatory proteins in this pathway, PDHA1, the catalytic subunit of the PDHC complex, emerged as a key protein of interest [19]. We found that PDHC activity was elevated in senescent PVECs from aged mice (Fig. 2A). This elevated activity was also evident in D-gal-induced senescence models in both PVECs (Fig. S3A) and HUVECs (Fig. S3D). Consistent with this finding, phosphorylation of PDHA1 at the inhibitory Ser293 site was markedly reduced in aged PVECs (Fig. 2B and C) and in D-gal-induced senescent PVECs (Fig. S3B and C) and HUVECs (Fig. S3E and F). This posttranslational modification (PTM) is an established mechanism for negative regulation of PDHC activity. In contrast, phosphorylation levels at other known sites, Ser300 and Ser232, remained unchanged in aged PVECs (Fig. 2B and C), highlighting the specificity of the Ser293 regulation. Previous studies demonstrated that pulmonary microvascular EC senescence plays a critical role in the pathogenesis of age-related COPD [24] and PF [25]. Immunofluorescence staining analysis revealed colocalization of the senescence-associated protein p21 and the endothelial marker CD31 in the lung tissues of aged mice (Fig. 2D and E), as well as in patients with COPD (Fig. 2F and G) or idiopathic PF (Fig. 2H and I), confirming senescence in these ECs. This senescent status was consistently associated with reduced phosphorylation of PDHA1 at Ser293 (Fig. 2D to I), which suggests an enhancement of PDHA1 activity in senescent pulmonary ECs.

### *Pdha1* depletion in ECs protects against D-gal-induced aging-related behavioral deficits in mice

**Fig. 3.**
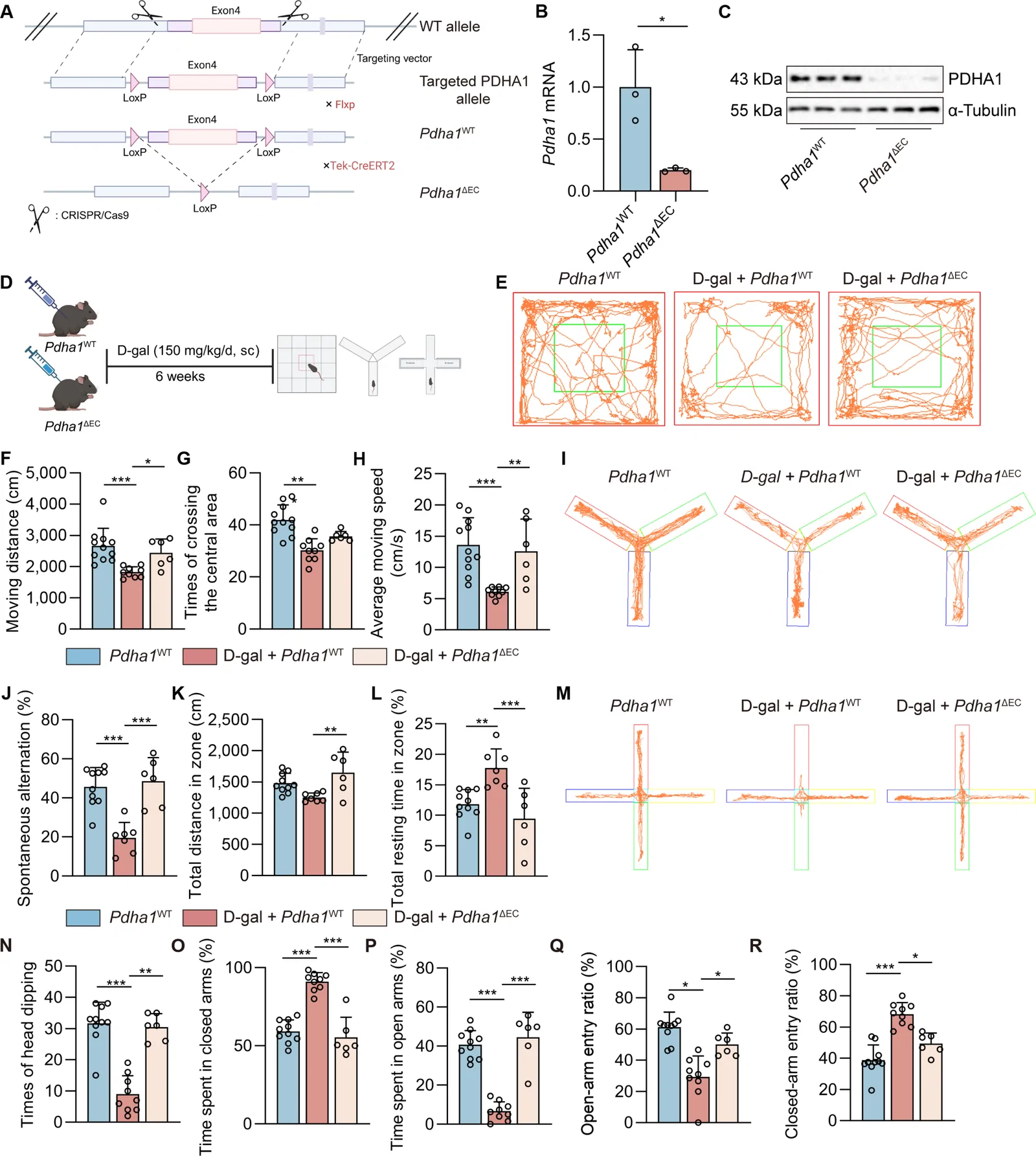
Endothelial-specific deletion of *Pdha1* and its alleviating effects on D-galactose (D-gal)-induced aging-related cognitive impairment. (A) Schematic of endothelial-specific *Pdha1*-null mouse generation. Exon4 was floxed in the targeted allele and excised in the conditional knockout allele. *Pdha1*^WT^ (*Ert2*-Cre-*Pdha1*^fl/fl^) and *Pdha1*^ΔEC^ (*Pdha1*^fl/fl^/*Ert*^Cre^). (B and C) Real-time polymerase chain reaction (PCR) and Western blot were used to detect pyruvate dehydrogenase E1 component subunit alpha (PDHA1) expression in pulmonary vascular endothelial cells (PVECs) from *Pdha1*^WT^ and *Pdha1*^ΔEC^ mice. (D) Mice were given a daily subcutaneous injection of D-gal (150 mg/kg) in the neck and back to establish the aging model. Following 6 weeks of treatment, the mice underwent the open-field test (OFT), Y-maze test, and elevated plus maze (EPM) test. (E) Movement trajectory of the mice in the OFT. (F to H) Moving distance, times of crossing the central area, and average moving speed during the OFT. (I) Representative trajectories of each group in the Y-maze test. (J to L) Behavioral measures in the Y-maze, namely, spontaneous alternation, total distance traveled, and total resting time in the zone. (M) Movement trajectory of the mice in the EPM. (N to R) The EPM test was performed to assess anxiety-like behavior, quantified by the number of head dips, the percentage of time spent in the closed arms, the percentage of time spent in the open arms, the number of open-arm entries, and the number of closed-arm entries. *n* = 6 to 10. *P < 0.05, **P < 0.01, and ***P < 0.001.

Endothelial-specific *Pdha1* knockout mice were created by crossing *Pdha1* floxed mice with Ert-Cre mice (Fig. 3A and Fig. S4A and C) to investigate the role of PDHA1 in endothelial senescence in vivo. We confirmed that PDHA1 expression was specifically deleted in PVECs isolated from *Pdha1*^fl/fl^/*Ert*^Cre^ (*Pdha1*^ΔEC^) mice, while regular expression was maintained in control *Ert*-Cre-*Pdha1*^fl/fl^ mice (*Pdha1*^WT^) (Fig. 3B and C). Furthermore, we verified that PDHA1 expression was not deleted in alveolar epithelial cells or macrophages in *Pdha1*^ΔEC^ mice (Fig. S4D and E). PDHC activity decreased in mouse primary PVECs isolated from *Pdha1*^ΔEC^ (Fig. S4B). To validate the aging model across multiple dimensions and provide additional evidence that endothelial PDHA1 plays a crucial role in systemic aging, we induced accelerated aging in mice via chronic D-gal injection (Fig. 3D). The aging process in mice is frequently accompanied by increased anxiety-like behavior, serving as a functional readout for neurological and systemic decline [26]. In the open-field test (OFT), D-gal challenge induced behavioral deficits such as reduced central area crossings, total distance, and average speed. However, the endothelial-specific deletion of *Pdha1* exerted a protective effect, as these deficits were attenuated in *Pdha1*^ΔEC^ mice relative to those in *Pdha1*^WT^ littermates (Fig. 3E to H). Spatial working memory was evaluated using the Y-maze test. Representative movement paths for each group are displayed in Fig. 3I. Notably, *Pdha1*^ΔEC^ mice exhibited a marked reversal of D-gal-induced cognitive impairment, demonstrating increased spontaneous alternation (Fig. 3J), greater total distance traveled (Fig. 3K), and total resting time in the zone (Fig. 3L). The anxiety-like behavior was further assessed using an elevated plus maze (EPM). Representative movement traces are shown in Fig. 3M. Compared with D-gal-induced *Pdha1*^WT^, *Pdha1*^ΔEC^ mice displayed elevated exploratory and reduced avoidance behavior, including more head-dipping episodes (Fig. 3N), decreased closed-arm time (Fig. 3O) and entries (Fig. 3P), and increased open-arm time (Fig. 3Q) and entries (Fig. 3R), consistent with an anxiolytic effect. Collectively, these behavioral findings demonstrate that the endothelial-specific deletion of *Pdha1* mitigates D-gal-induced, aging-related cognitive decline and anxiety-like behavior.

### Endothelial-specific *Pdha1* deletion attenuates pulmonary endothelial senescence in vivo

**Fig. 4.**
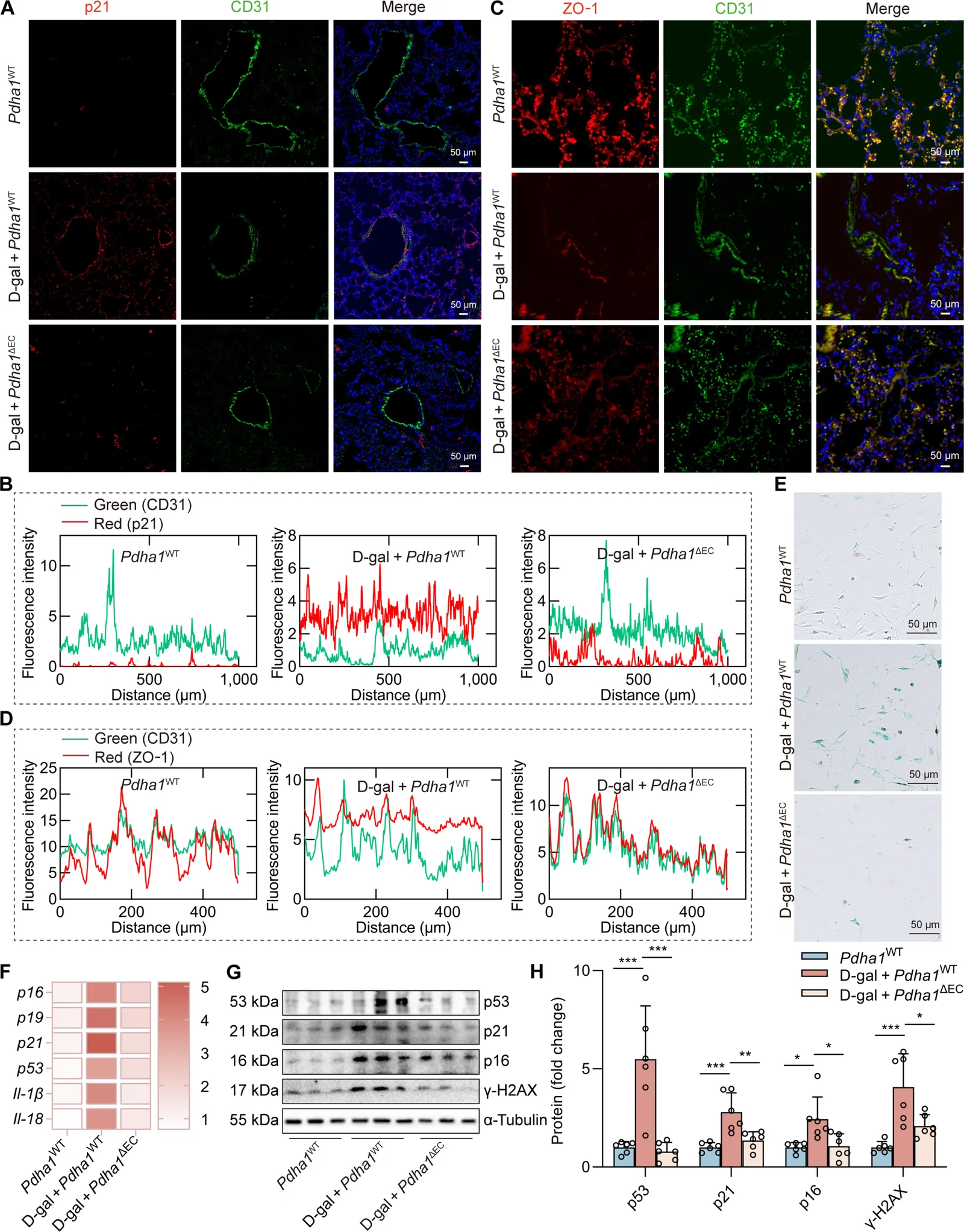
Endothelial-specific deletion of *Pdha1* attenuates senescence in pulmonary vascular endothelial cells (PVECs) in vivo. *Pdha1*^WT^ and *Pdha1*^ΔEC^ mice were given a daily subcutaneous injection of D-galactose (D-gal; 150 mg/kg) in the neck for 6 weeks. (A) The fluorescence intensity of p21 (red) and CD31 (green) was assessed by immunofluorescence staining (IF) in lungs obtained from both young and D-gal-induced aged mice. Scale bar = 50 μm. (B) The fluorescence intensity curve shows the distribution of p21 (red) and CD31 (green) along the cells. (C) The colocalization of zonula occludens-1 (ZO-1; red) and CD31 (green) in lungs from mice with D-gal-induced aged mice was detected by IF. Scale bar = 50 μm. (D) The fluorescence intensity curve shows the distribution of ZO-1 (red) and CD31 (green) along the cells. (E) Senescence-associated β-galactosidase (SA-β-gal) staining was assessed in mouse primary PVECs isolated from *Pdha1*^WT^ and *Pdha1*^ΔEC^ mice. Scale bar = 50 μm. (F) Senescence-related marker gene expression, including *p16*, *p19*, *p21*, *p53*, *Il-1β*, and *Il-18* messenger RNA (mRNA) in mouse primary PVECs isolated from *Pdha1*^WT^ and *Pdha1*^ΔEC^ mice, was detected by real-time polymerase chain reaction (PCR). (G and H) Western blot was used to detect p53, p21, p16, and phosphorylated histone H2AX (γ-H2AX) expression in mouse primary PVECs isolated from *Pdha1*^WT^ and *Pdha1*^ΔEC^ mice. *n* = 6. *P < 0.05, **P < 0.01, and ***P < 0.001.

In addition, *Pdha1*^ΔEC^ ameliorated D-gal-induced pulmonary structural abnormalities, attenuated alveolar destruction, and suppressed airspace enlargement (Fig. S5). Immunofluorescence analysis revealed colocalization of the senescence marker p21 with the endothelial marker CD31 in the lung tissues of D-gal-induced aged mice (Fig. 4A), indicating the presence of senescent ECs. Notably, *Pdha1*^ΔEC^ mice exhibited decreased endothelial (CD31^+^) senescence-related molecule p21 expression (Fig. 4A and B). Furthermore, endothelial-specific *Pdha1* deletion conferred protection against aging-induced barrier disruption, supported by the enhanced continuity of zonula occludens-1 (ZO-1) staining in *Pdha1*^ΔEC^ mouse lungs relative to that *Pdha1*^WT^ controls (Fig. 4C and D). Then, PVECs were isolated from *Pdha1*^WT^ and *Pdha1*^ΔEC^ mice. The number of SA-β-gal-positive cells was markedly reduced in PVECs following endothelial-specific *Pdha1* deletion and D-gal induction (Fig. 4E and Fig. S14A). Correspondingly, the mRNA levels of senescence-associated markers (*p16*, *p19*, *p21*, *p53*, *Il-1β*, and *Il-18*) were down-regulated in PVECs from *Pdha1*^ΔEC^ mice relative to those from *Pdha1*^WT^ mice after D-gal induction (Fig. 4F). Western blot analysis revealed a decrease in the levels of senescence-related proteins, such as p53, p21, p16, and γ-H2AX, in PVECs from *Pdha1*^ΔEC^ mice compared with those in *Pdha1*^WT^ mice (Fig. 4G and H). These results establish PDHA1 as a critical driver of pulmonary EC senescence.

### PDHA1-dependent metabolic reprogramming orchestrates PVECs’ senescence in vitro

**Fig. 5.**
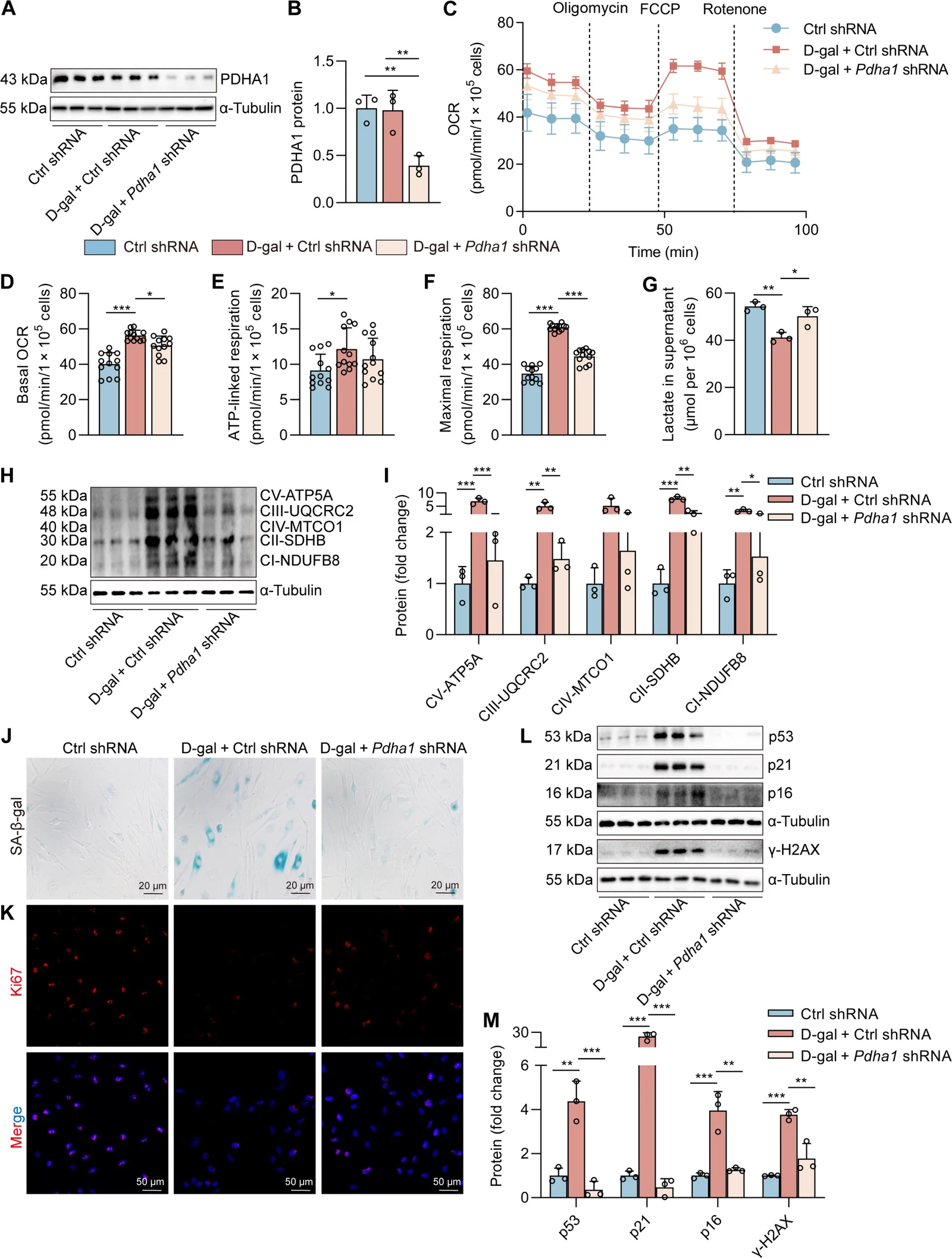
*Pdha1* deficiency attenuates cellular senescence by reversing metabolic reprogramming in vitro. Pulmonary vascular endothelial cells (PVECs) were treated with Ctrl short hairpin RNA (shRNA) or *Pdha1* shRNA for 4 to 6 h, followed by 48-h incubation in a complete medium, and then exposed to D-gal (30 mg/ml). Cell samples were harvested after 24 h. (A and B) Western blot for pyruvate dehydrogenase E1 component subunit alpha (PDHA1) protein expression in *Pdha1* shRNA-treated PVECs. (C and D) The oxygen consumption rate (OCR) was obtained using Seahorse XF24 Analyzer. (E and F) Statistical analyses of adenosine triphosphate (ATP)-linked respiration and maximal respiration in *Pdha1* shRNA-treated PVECs. (G) Analysis of lactate production. (H and I) The expression of oxidative phosphorylation (OXPHOS)-related proteins (ATP5A, UQCRC2, MTCO1, SDHB, and NDUFB8) in PVECs treated with *Pdha1* shRNA was detected by Western blot analysis. *n* = 3. (J and K) Senescence-associated β-galactosidase (SA-β-gal) staining (scale bar = 20 μm) and immunofluorescence staining (IF) for Ki67 (scale bar = 50 μm) were assessed in PVECs treated with *Pdha1* shRNA. (L and M) Western blot for p53, p21, p16, and phosphorylated histone H2AX (γ-H2AX) protein expression in *Pdha1* shRNA-treated PVECs. α-Tubulin was used as the internal control. *n* = 3. *P < 0.05, **P < 0.01, and ***P < 0.001.

To investigate the metabolic mechanism by which PDHA1 drives endothelial senescence, we conducted genetic silencing experiments in vitro (Fig. 5A and B). Consequently, *Pdha1* silencing attenuated the D-gal-induced increases in OCR (Fig. 5C and D), ATP-linked respiration (Fig. 5E), and maximal respiration (Fig. 5F). Consistent with these functional alterations, Western blot analysis demonstrated that the up-regulation of OXPHOS-related proteins (ATP5A, UQCRC2, MTCO1, SDHB, and NDUFB8) induced by D-gal was also reversed upon *Pdha1* silencing in PVECs (Fig. 5H and I). *Pdha1* silencing in PVECs exhibited increased D-gal-induced lactate production (Fig. 5G). Collectively, these findings indicate that PDHA1 is critical for driving the metabolic shift toward enhanced OXPHOS and away from glycolytic activity in senescent PVECs. Furthermore, we found that *Pdha1* silencing reduced the population of D-gal-induced β-gal-positive PVECs and increased levels of the cell proliferation marker Ki67 (Fig. 5J and K and Fig. S14B). Concomitantly, the intervention consequently inhibited the expression of key senescence-associated proteins, including p53, p21, p16, and γ-H2AX (Fig. 5L and M). Similarly, competitive inhibition of PDHA1 with 3-fluoropyruvate (3-FP) [27] also phenocopied the effects of *Pdha1* silencing, attenuating the senescence phenotype by reducing the D-gal-induced increase in β-gal-positive cells (Fig. S6A and B) and the levels of key senescence markers (p53, p21, p16, and γ-H2AX) (Fig. S6C and D). These data demonstrate that *Pdha1* silencing attenuates PVEC senescence, suggesting that PDHA1-dependent metabolic alterations may act as a driving force for senescence in pulmonary ECs.

### PDHA1^S293^ dephosphorylation-induced PDHA1 hyperactivation promotes senescence in ECs

**Fig. 6.**
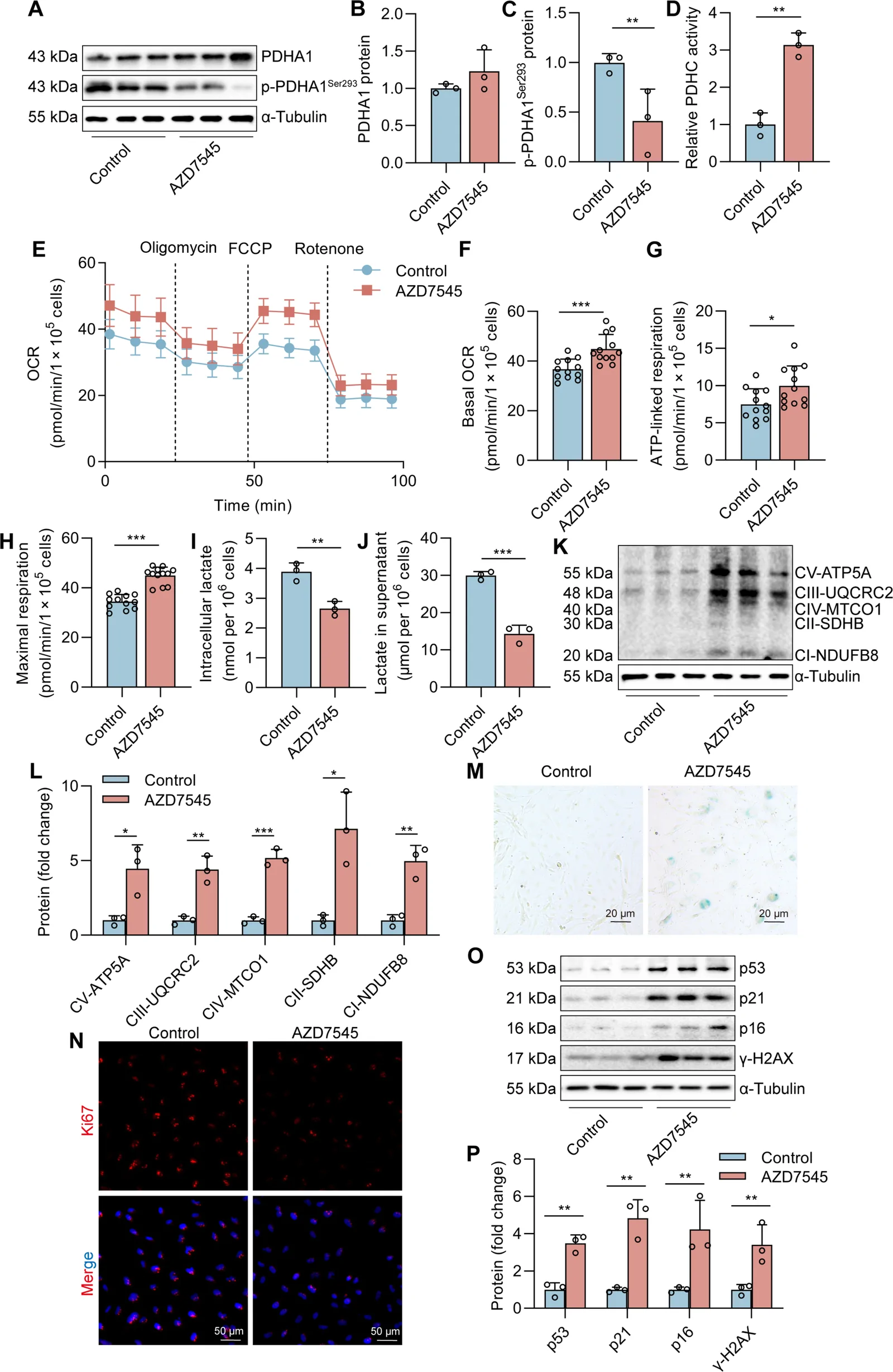
Pyruvate dehydrogenase E1 component subunit alpha (PDHA1) activation effectively accelerates senescence in endothelial cells. Pulmonary vascular endothelial cells (PVECs) were treated with the PDHA1 activator AZD7545 (40 μM) for 24 h. (A to C) Western blot for PDHA1 and p-PDHA1^Ser293^ in AZD7545-treated PVECs; *n* = 3. (D) Pyruvate dehydrogenase complex (PDHC) activity was measured by a commercially available PDHC activity assay kit. (E and F) The oxygen consumption rate (OCR) was obtained using Seahorse XF24 Analyzer. (G and H) Statistical analyses of adenosine triphosphate (ATP)-linked respiration and maximal respiration in AZD7545-treated PVECs. (I and J) Analysis of lactate production in the cytosol and culture supernatant. (K and L) The expression of oxidative phosphorylation (OXPHOS)-related proteins (ATP5A, UQCRC2, MTCO1, SDHB, and NDUFB8) in PVECs treated with AZD7545 was detected by Western blot analysis. *n* = 3. (M) Senescence-associated β-galactosidase (SA-β-gal) staining was assessed in the PVECs treated with AZD7545 (scale bar = 20 μm). (N) Immunofluorescence for Ki67 protein expression in AZD7545-treated PVECs; scale bar = 50 μm. (O and P) Western blot for p53, p21, p16, and phosphorylated histone H2AX (γ-H2AX) protein expression in AZD7545-treated PVECs. α-Tubulin was used as the internal control. *n* = 3. *P < 0.05, **P < 0.01, and ***P < 0.001.

To determine whether PDHA1 activation alone is sufficient to promote endothelial senescence, we treated PVECs with AZD7545, a pharmacological inhibitor of pyruvate dehydrogenase kinase (PDK) that promotes PDHA1 dephosphorylation and activation [28]. AZD7545 treatment markedly reduced phosphorylation at the Ser293 site of PDHA1 (Fig. 6A to C). Consequently, PDHC activity was enhanced in PVECs, an effect that was inversely correlated with the level of S293 phosphorylation (Fig. 6D). Treatment with AZD7545 enhanced mitochondrial function in ECs, as evidenced by increased OCR, ATP-linked respiration, and maximal respiration (Fig. 6E to H). Consistent with these functional improvements, AZD7545 up-regulated the expression of OXPHOS-related proteins (ATP5A, UQCRC2, MTCO1, SDHB, and NDUFB8) in PVECs (Fig. 6K and L), while it concurrently decreased lactate production in PVECs (Fig. 6I and J). Collectively, these findings indicate that PDHA1 activation drives a metabolic shift from glycolysis to OXPHOS. Furthermore, PDHA1 activation promoted the senescent phenotype, which was demonstrated by an increase in the proportion of SA-β-gal-positive PVECs (Fig. 6M and Fig. S14C), decreased levels of the proliferation marker Ki67 (Fig. 6N), and up-regulation of key senescence-associated proteins, including p53, p21, p16, and γ-H2AX (Fig. 6O and P). However, AZD7545 alone at 20 μM did not appreciably up-regulate senescence markers, whereas its combination with a subeffective dose of D-gal (15 mg/ml) enhanced the senescent phenotype (Fig. S7A to F), indicating that additional PDHA1 activation in the D-gal background produces further prosenescent effects. To validate the role of OXPHOS in PDHA1-activation-induced senescence, ECs were treated with the ATP synthase inhibitor oligomycin alongside AZD7545. Oligomycin reversed the AZD7545-induced up-regulation of p53, p21, p16, and γ-H2AX (Fig. S8A to E), indicating that the prosenescent effect of PDHA1 activation depends on enhanced OXPHOS. Our findings demonstrate that reduced phosphorylation of PDHA1 at Ser293 was consistently associated with endothelial senescence. Flow cytometry confirmed that the purity of ECs following PDHA1-S293A mutant treatment was approximately 85% (Fig. S9A). Overexpression of a phosphodeficient PDHA1-S293A mutant enhanced PDHA1 activation (Fig. S9B) and triggered a robust senescent phenotype in primary murine PVECs, characterized by enhanced SA-β-gal activity (Fig. S9C and D) and coordinated up-regulation of key senescence-related and DNA damage markers (Fig. S9E and F). In summary, PDHA1^S293^ dephosphorylation-induced hyperactivation initiates a metabolic reprogramming from glycolysis toward OXPHOS, which in turn induces EC senescence.

### Decreased lactylation of PDHA1 at Lys336 facilitates its dephosphorylation and drives PVECs’ senescence

**Fig. 7.**
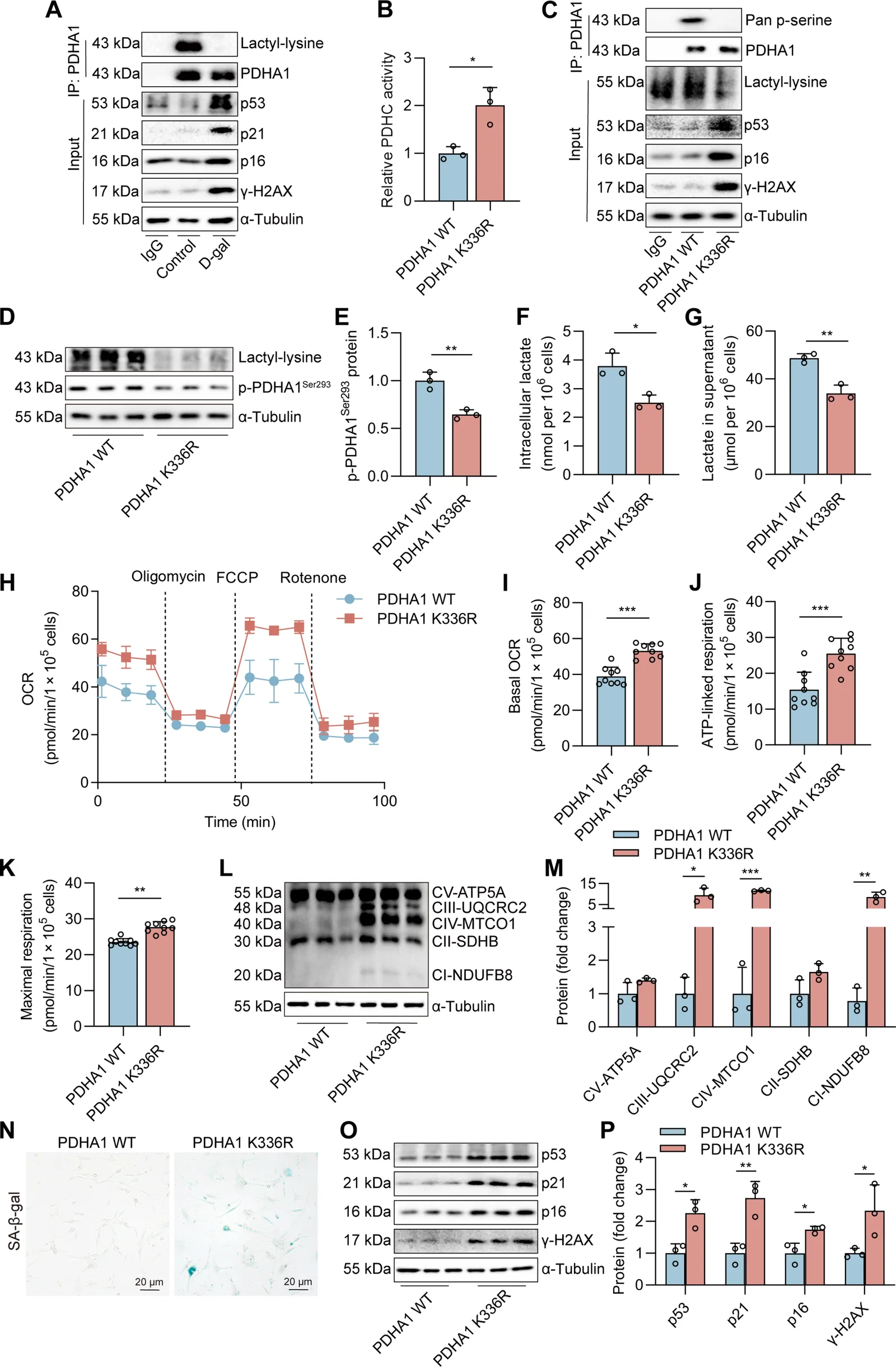
Decreased lactylation of pyruvate dehydrogenase E1 component subunit alpha (PDHA1) at Lys336 facilitates its dephosphorylation and drives a metabolic shift. (A) Pulmonary vascular endothelial cells (PVECs) were treated with D-galactose (D-gal; 30 mg/ml) for 24 h. Cell lysates were immunoprecipitated with PDHA1 antibody-conjugated Protein A/G Agarose, followed by Western blot for the lactylation of PDHA1 using anti-pan-lactyl antibodies. (B) PDHA1-K336R mutation increased pyruvate dehydrogenase complex (PDHC) activity in mouse primary PVECs. (C) Primary murine PVECs were cotransfected with wild-type PDHA1 WT or PDHA1-K336R mutant. Cell lysates were immunoprecipitated with PDHA1 antibody-conjugated Protein A/G Agarose, followed by a Western blot for the phosphorylation of PDHA1 using pan p-serine antibodies. (D and E) Lactyl-lysine protein level and the phosphorylation level at PDHA1 S293 were determined. (F and G) Quantification of intracellular and extracellular lactate levels. (H and I) The oxygen consumption rate (OCR) of PVECs transfected with the PDHA1-K336R mutant was measured using Seahorse XF24 Analyzer. (J and K) Statistical analyses of adenosine triphosphate (ATP)-linked respiration and maximal respiration. (L and M) The expression of oxidative phosphorylation (OXPHOS)-related proteins in PVECs transfected with the PDHA1-K336R mutant was detected by Western blot analysis. (N) Senescence-associated β-galactosidase (SA-β-gal) staining was assessed in mouse primary PVECs with PDHA1 WT or PDHA1 K336R (scale bar = 20 μm). (O and P) p53, p21, p16, and phosphorylated histone H2AX (γ-H2AX) protein levels in PVECs with PDHA1 WT or PDHA1 K336R were detected by Western blot. *n* = 3. *P < 0.05, **P < 0.01, and ***P < 0.001.

We next investigated the mechanism underlying the hyperactivation of PDHA1 in senescent PVECs. Although PDHA1 activity is known to be catalyzed primarily by PDK1 [29], we found that the protein level of this canonical regulatory enzyme was not significantly altered in senescent PVECs (Fig. S10A to D). Lactate serves as the substrate for protein lactylation. We found a decrease in global protein lactylation in both naturally aged PVECs (Fig. S11A) and the D-gal-induced model of cellular senescence in PVECs (Fig. S11B). Pretreatment with lactate further alleviated D-gal-induced senescent phenotype (Fig. S11C to H), suggesting that reduced lactylation contributes to endothelial aging. Coimmunoprecipitation (Co-IP) showed that PDHA1 lactylation was markedly reduced in D-gal-induced senescent PVECs (Fig. 7A). Motivated by reported lactylation at PDHA1 K336 [30], we expressed PDHA1 K336 to arginine (R) in murine PVECs and observed increased PDHC activity (Fig. 7B). Given the central role of phosphorylation in PDHA1 regulation, we further assessed the functional cross talk between lactylation and phosphorylation. Expression of the delactylase-inactive PDHA1-K336R mutant markedly reduced global PDHA1 phosphorylation (Fig. 7C), with a pronounced dephosphorylation specifically at the inhibitory Ser293 site (Fig. 7D and E). We further confirmed the regulatory interplay by showing that lactate supplementation promoted PDHA1 phosphorylation at Ser293 (Fig. S11I and J) and consequently suppressed PDHC activity upon D-gal challenge (Fig. S11K). These findings collectively indicate that lactylation at Lys336 allosterically restricts phosphorylation at Ser293, thereby modulating PDHA1 function. We further examined the functional consequences of reduced PDHA1 lactylation. Overexpression of the delactylase-inactive PDHA1-K336R mutant promoted a metabolic shift from glycolysis toward oxidative glucose metabolism, as reflected by a reduction in the glycolysis end-product lactate (Fig. 7F and G), along with increases in OCR (Fig. 7H and I), ATP-linked respiration (Fig. 7J), and maximal respiration (Fig. 7K). At the molecular level, this shift was further supported by the up-regulation of a suite of OXPHOS-related proteins, ATP5A, UQCRC2, MTCO1, SDHB, and NDUFB8, in PVECs (Fig. 7L and M). Furthermore, the PDHA1-K336R mutant induced premature senescence in primary murine PVECs, characterized by an increase in SA-β-gal-positive cells (Fig. 7N and Fig. S14D), concomitant with the up-regulation of senescence markers p53, p21, p16, and γ-H2AX (Fig. 7O and P). To further confirm the role of lactylation modification in the D-gal pathological context, we overexpressed PDHA1 K336R in D-gal-treated PVECs and found that it further exacerbated the up-regulation of senescence markers on top of D-gal-induced senescence (Fig. S12A to E). Collectively, these findings suggest that decreased lactylation of PDHA1 facilitates its dephosphorylation and promotes a metabolic shift, thereby establishing a novel mechanism for EC senescence.

### PDHA1 activation drives mtDNA–cGAS–STING-mediated endothelial senescence

**Fig. 8.**
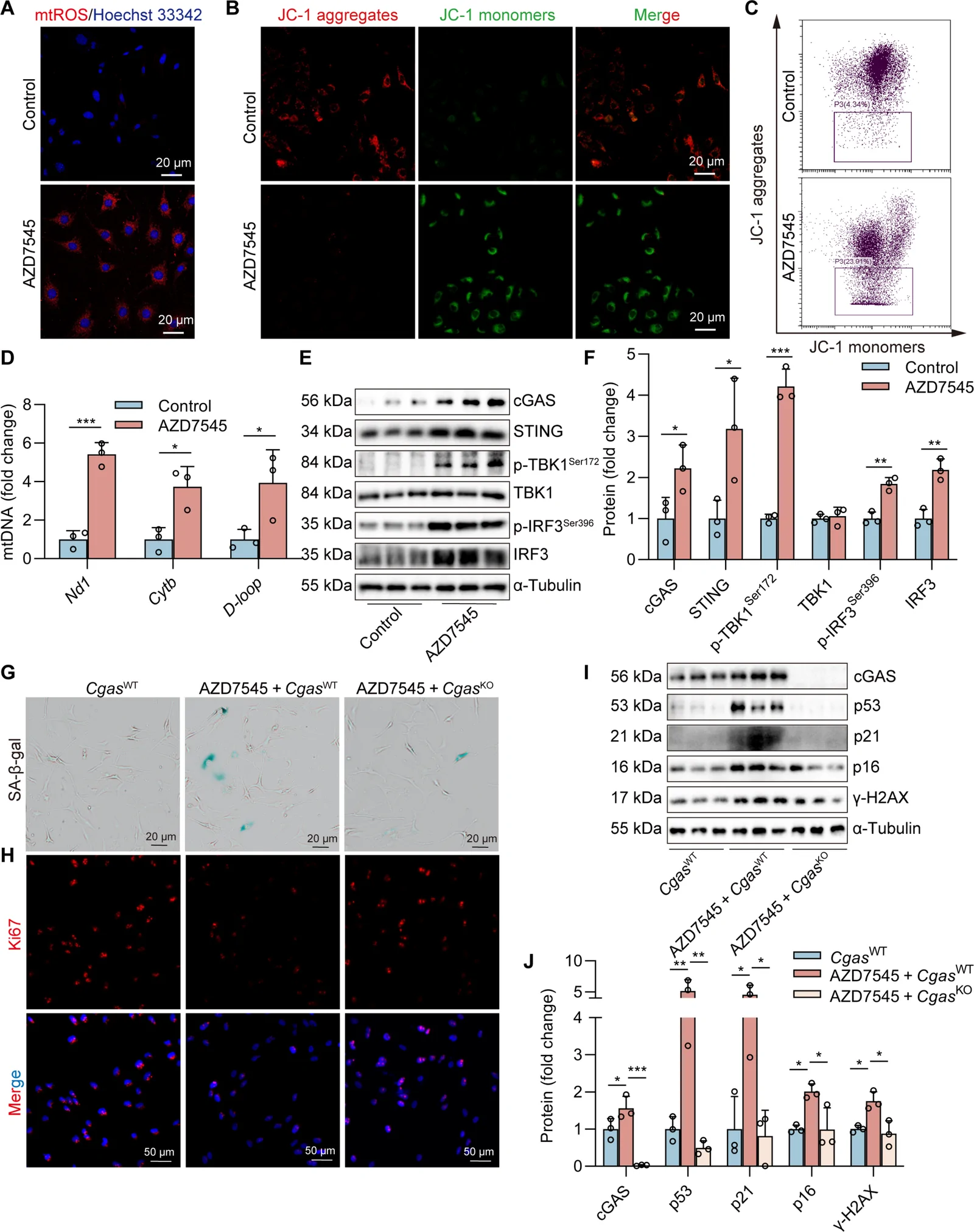
Pyruvate dehydrogenase E1 component subunit alpha (PDHA1)-activation-driven endothelial senescence via the cyclic GMP-AMP synthase (cGAS) signaling pathway. Cellular senescence in pulmonary vascular endothelial cells (PVECs) was induced by treatment with AZD7545. (A) The mitochondrial reactive oxygen species (mtROS) in AZD7545-treated PVECs were detected by an ROS kit (scale bar = 20 μm). (B and C) JC-1 staining showing mitochondrial membrane potential in endothelial cells (scale bar = 20 μm). (D) Quantification of cytosolic mitochondrial DNA (mtDNA) for the *Nd1*, *Cytb*, and *D-loop* in AZD7545-treated PVECs was performed by real-time polymerase chain reaction (PCR). (E and F) Western blot and quantification for cGAS, stimulator of interferon genes (STING), p-TBK1^Ser172^, TBK1, p-IRF3^Ser396^, and IRF3 protein expression. (G) Primary PVECs isolated from *Cgas*^WT^ and *Cgas*^KO^ mice were stimulated with AZD7545 (40 μM) for 24 h. Senescence-associated β-galactosidase (SA-β-gal) staining was performed to detect AZD7545-induced senescence in primary PVECs from *Cgas*^WT^ and *Cgas*^KO^ mice; scale bar = 20 μm. (H) Ki67 staining was performed in AZD7545-treated primary PVECs from *Cgas*^KO^ mice; scale bar = 50 μm. (I and J) The expression of senescence-related proteins (cGAS, p53, p21, p16, and phosphorylated histone H2AX [γ-H2AX]) was examined by Western blot in AZD7545-treated primary PVECs isolated from *Cgas*^WT^ and *Cgas*^KO^ mice. α-Tubulin was used as the internal control. *n* = 3. *P < 0.05, **P < 0.01, and ***P < 0.001.

We next investigated the mechanism by which PDHA1 hyperactivation promotes endothelial senescence. An overactive OXPHOS system places a substantial oxidative burden on mitochondria, resulting in excessive mitochondrial reactive oxygen species (mtROS). Our studies found that senescent PVECs induced by AZD7545 showed elevated mtROS levels (Fig. 8A) and concurrently dissipated mitochondrial membrane potential, evidenced by an altered JC-1 aggregate/monomer ratio (Fig. 8B and C). Similarly, pharmacological inhibition of PDHA1 attenuated D-gal-induced mitochondrial damage, with reduced mtROS production (Fig. S13A) and a preserved mitochondrial membrane potential (Fig. S13B and C). Since mtROS can cause mitochondrial DNA (mtDNA) oxidation and subsequent cytosolic release [31], we quantified cytosolic mtDNA and confirmed its accumulation in AZD7545-induced senescent PVECs (Fig. 8D). Collectively, these findings demonstrate that PDHA1 activation promotes senescence by inducing pronounced mitochondrial oxidative stress and dysfunction. The cGAS–STING pathway is a DNA-sensing signaling cascade that plays a pivotal role in driving cellular senescence. As shown in Fig. 8E, the cGAS–STING signaling pathway was activated upon PDHA1 activation, as demonstrated by the increased levels of cGAS, p-TBK1^Ser172^, and p-IRF3^Ser396^ (Fig. 8E and F). Correspondingly, the D-gal-induced activation of the cGAS–STING pathway was abolished by 3-FP, a specific pharmacological inhibitor of PDHA1 (Fig. S13D and E). To further determine whether cGAS–STING signaling contributes to PDHA1-hyperactivation-dependent senescence, we isolated primary PVECs from *Cgas*^WT^ and *Cgas*^KO^ mice. Genetic ablation of *Cgas* markedly attenuated the PDHA1-hyperactivation-induced senescent phenotype. This was supported by reduced SA-β-gal activity (Fig. 8G and Fig. S14E), an increase in Ki67-positive cells (indicating enhanced proliferation, Fig. 8H), and the down-regulation of key senescence-associated proteins, p53, p21, p16, and γ-H2AX (Fig. 8I and J). Taken together, these data indicate that PDHA1 hyperactivation disrupts mitochondrial homeostasis, producing excessive mtROS, which results in oxidative damage to mtDNA and its release into the cytoplasm while switching on cGAS–STING-mediated senescence in ECs.

## Discussion

**Fig. 9.**
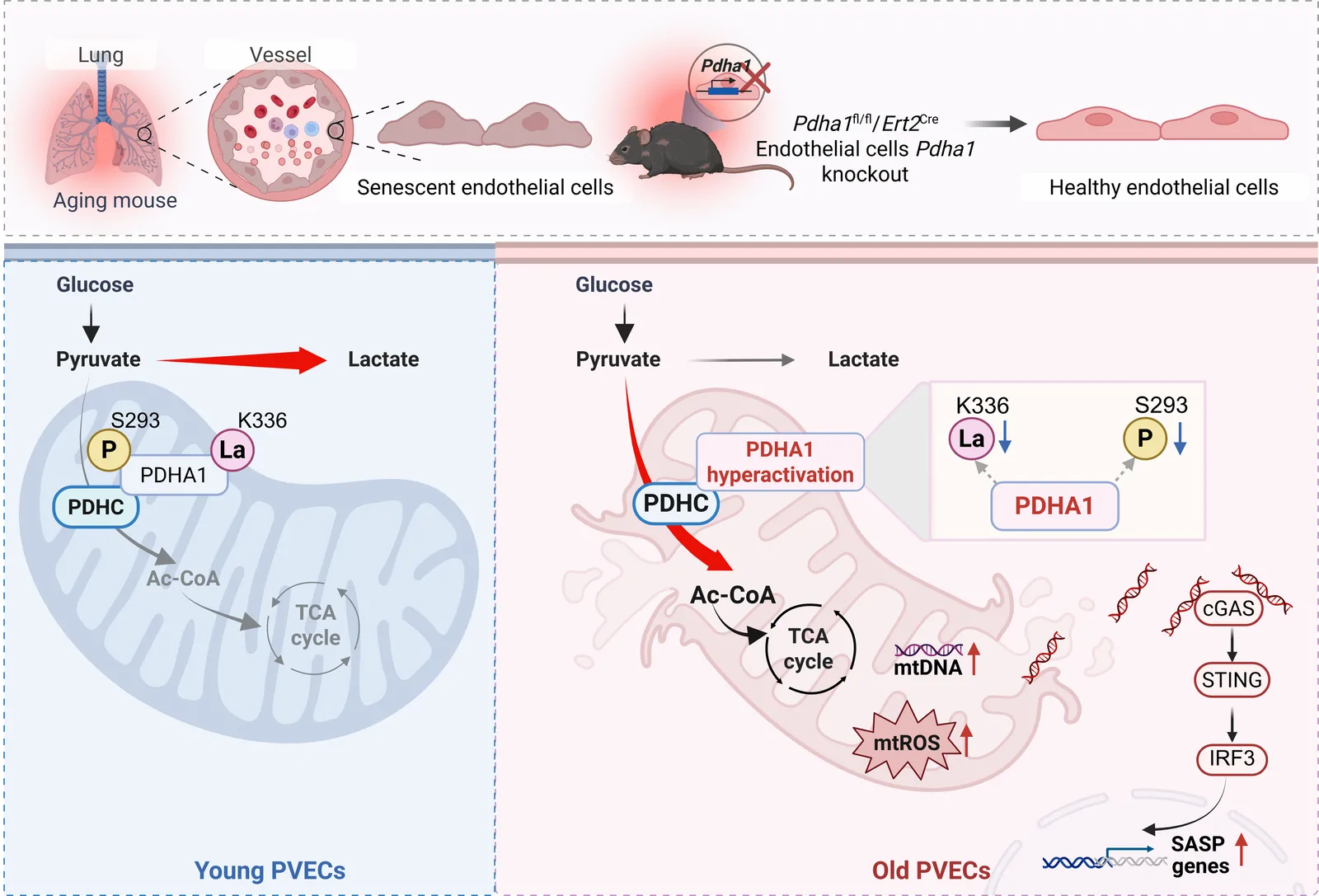
Schematic illustration. Pyruvate dehydrogenase E1 component subunit alpha (PDHA1) activation, driven by S293 dephosphorylation resulting from reduced lactylation at K336, disrupts mitochondrial homeostasis, leading to mitochondrial reactive oxygen species (mtROS)-mediated mitochondrial DNA (mtDNA) release and ultimately triggering cyclic GMP-AMP synthase–stimulator of interferon genes (cGAS–STING)-dependent senescence.

The senescence of pulmonary ECs is a pivotal driver in the pathogenesis of age-related lung diseases. Despite its clinical importance, the molecular underpinnings of this process remain poorly understood. In this study, we systematically characterized senescent phenotypes and targeted metabolomic profiles in primary aging ECs, identifying a PDHA1-dependent metabolic shift as a hallmark of senescence in these cells. Using a D-gal-induced senescence model, we demonstrated that endothelial-specific *Pdha1* knockdown via gene therapy mitigated pulmonary vascular senescence and associated functional decline. We further revealed that PDHA1-driven OXPHOS triggers mitochondrial dysfunction, leading to the subsequent activation of the cGAS–STING pathway and initiation of senescence-associated signaling. Mechanistically, PDHA1 activity is potentiated by reduced lactylation at K336, which promotes S293 dephosphorylation. Together, our results identify PDHA1 hyperactivation as a previously unrecognized driver of endothelial senescence (Fig. 9).

Our findings indicate that both naturally aged and D-gal-induced senescent PVECs up-regulate oxidative glucose metabolism while restricting glycolytic flux. Our previous findings indicate that glycolysis-dominant metabolic reprogramming during acute inflammation promotes the development of lung injury [10,32]. It is noteworthy that mitochondrial impairment and reduced OXPHOS are typically considered hallmarks of the senescent phenotype [33]. The enhanced respiratory activity may be attributed to increased PDHA1 function, as suggested by its reduced phosphorylation at Ser293, which facilitates greater pyruvate entry into the TCA cycle via acetyl-CoA. Among the key phosphorylation sites, Ser293 exhibited the fastest kinetics and the most potent deactivating effect, whereas phosphorylation at Ser300 and Ser232 occurred more slowly and resulted in weaker inactivation [34,35]. Consequently, Ser293 is established as the predominant site for the phospho-regulation of PDH activity [36]. Our work implies that endothelial senescent status was consistently associated with reduced phosphorylation of PDHA1 at Ser293. These results reveal an unexpected up-regulation of OXPHOS components in endothelial senescence. This apparent metabolic paradox can be reconciled by a compensatory mechanism [37,38]. mtDNA mutations and deletions accumulate with age; cells can enhance the transcription of unaffected mitochondrial genes and up-regulate OXPHOS complex expression. Indeed, senescent human ECs exhibit increased levels of ND2, ND3, ATPase 6, and 16S rRNA transcript [39]. Moreover, in oncogene-induced senescence, PDH activation drives pyruvate into the TCA cycle, increasing oxygen consumption and reactive oxygen species, a metabolic reprogramming critical for executing the senescence program [40]. Furthermore, age-related bioenergetic changes are context dependent; healthy older individuals or animals may exhibit higher respiratory capacity than those with functional decline or chronic disease [41]. Mechanistically, PDHA1 hyperactivation increases acetyl-CoA supply, alters NAD^+^/NADH and ATP/AMP ratios, and activates the AMPK–SIRT1–PGC-1α axis, up-regulating mitochondrial biogenesis regulators [42–44]. The high expression of mitochondrial respiratory chain complexes I and III leads to a marked increase in mtROS levels [45], and elevated mtROS promote senescence via mtDNA damage and induction of p21/p16 [46]. mtROS are also known to propagate organellar dysfunction in a self-amplifying manner [47], which may elicit a compensatory rise in mitochondrial biogenesis [48], coupled with suppressed mitophagy, ultimately leading to the accumulation of damaged mitochondria [49]. Thus, the elevated OXPHOS observed in our model represents a programmed, compensatory high-metabolic-stress state during active senescence execution rather than a genuine long-term functional enhancement. Sustained high-flux operation and accumulated oxidative stress may eventually lead to functional decline.

Our findings establish that endothelial-specific *Pdha1* deletion orchestrates a metabolic reprogramming toward glycolysis, which unexpectedly confers protection against cellular senescence and age-related cognitive decline. Seahorse analysis revealed a distinctive metabolic profile characterized by suppressed glycolytic capacity yet elevated lactate release, coupled with enhanced mitochondrial respiratory parameters. These observations align with reports in C2C12 cells [30] and extend earlier findings in HUVECs [50], in which PDHC inhibitor-induced metabolic shift to oxidative metabolism suppressed EC proliferation and migration. However, that study did not assess the potential link to cellular senescence. Our data establish the transition to an oxidative metabolic state as a driver of cellular senescence. Our detection of heightened PDHA1 activity in patient lung tissues reveals that it drives EC senescence to promote disease progression. Endothelial *Pdha1* knockout ameliorated D-gal-induced lung pathology and aging-related cognitive decline, validating that endothelial PDHA1 plays a crucial role in systemic aging. Future studies will incorporate measurements such as tidal volume, minute ventilation, airway resistance, or pulse oximetry to monitor lung function dynamically. This finding broadens our earlier hypothesis, which was primarily focused on senescence as a key driver of PF [51–53]. This finding further corroborates the established function of PDHA1 in modulating oncogene-induced senescence [54]. This phenomenon may be attributed not only to increased pyruvate flux into oxidative pathways but also to a diminished NAD^+^/NADH ratio, resulting from heightened NAD^+^ consumption by the PDH reaction coupled with insufficient NAD^+^ regeneration [55]. Cells treated with *Pdha1* activity exhibited elevated mtROS production and an increase in mitochondrial mass, indicative of organelle dysfunction. This may lead to the leakage of mtDNA into the cytosol, thereby activating the canonical cGAS–STING pathway of cellular senescence. We previously demonstrated that sevoflurane potentiates DRP1-dependent mitochondrial fission, facilitating mtDNA release into the cytosol through the MPTP–VDAC complex. This cytosolic mtDNA subsequently activates the cGAS–STING signaling pathway, triggering NLRP3 inflammasome assembly and ultimately contributing to postoperative cognitive dysfunction [56] and pulmonary inflammation [57]. The established role of cGAS–STING activation in driving endothelial senescence in early diabetic retinopathy [58] is complemented by its parallel involvement in aging-associated endothelial dysfunction [59], collectively underscoring the fundamental importance of this pathway across diverse vascular degenerative conditions.

It is well established that PDHA1 is inactivated upon phosphorylation by PDK [60]. However, we found that the PDK protein level of this canonical regulatory enzyme was not altered in senescent PVECs. In recent years, accumulating evidence has highlighted the presence of cross talk among various PTMs [61,62]. Such PTMs can either physically interfere with one another or act cooperatively to initiate or suppress downstream signaling, thereby executing specific biological functions [61]. In this study, we found that decreased lactylation of PDHA1 at lysine 336 promotes dephosphorylation at Ser293. This modification attenuates a downstream metabolic shift that, when hyperactive, triggers mtROS production and subsequent mtDNA leakage into the cytosol, ultimately activating the canonical cGAS–STING pathway linked to cellular senescence. Lysine lactylation, a recently identified PTM, is increasingly recognized as a regulator of cellular processes [63]. Our study elucidates how reduced lactylation of PDHA1 interacts with canonical phosphorylation events to regulate its kinase activity, providing new insights into the complex metabolic regulation underlying endothelial senescence. Previous reports show that under aerobic conditions, PDHA1 lactylation enhances lactate production and fatty acid oxidation in mouse muscle [64], whereas under hypoxia, it decreases PDHA1 lactylation to promote OXPHOS [30]. Positive cross talk between phosphorylation and SUMOylation has been established in key cancer-related proteins. For instance, in the case of p53, phosphorylation at Ser18 promotes SUMOylation mediated by Ubc9, leading to p53 stabilization and suppression of pancreatic β-cell proliferation [65]. In such positive cross talk scenarios, one PTM may either recruit enzymes responsible for another modification or create a binding interface for effector proteins that execute the functions of a secondary PTM [66]. A recent seminal study established that the disruption of the lactate–H3K18la axis underlies TRPM7 deficiency-induced vascular senescence, highlighting the role of reduced lactylation in endothelial aging [63]. This discovery aligns with and strongly supports our central finding that decreased lactylation of PDHA1 serves as a novel metabolic trigger driving endothelial senescence, thereby confirming decreased lactylation as an emerging hallmark of vascular aging.

This work invites due consideration of its inherent constraints. The precise mechanism by which PDHA1 hyperactivation promotes EC senescence remains unclear. Our metabolomics data revealed a marked accumulation of succinate and citrate in senescent ECs. Previous studies have indicated that succinate accumulation contributes to mitochondrial dysfunction under ischemic conditions [67], and up-regulated succinylation modifications can induce a senescent phenotype in microglia by disrupting mitochondrial energy metabolism [68]. However, it is still unknown whether PDHA1 hyperactivation drives succinate buildup, leading to mitochondrial impairment, subsequent mtDNA leakage, and ultimately cGAS-dependent senescence in ECs.

## Conclusion

Collectively, our study demonstrated that PDHA1-hyperactivation-dependent metabolic shift is a critical driver of EC senescence. Mechanistically, we reveal a previously unrecognized pathway in which decreased lactylation of PDHA1 facilitates its dephosphorylation, prompting a metabolic shift that ultimately promotes senescence. This study thus unveils a new regulatory mechanism in aging endothelium, proposing the targeting of PDHA1 hyperactivation as a viable strategy against vascular senescence.

## Materials and Methods

### Aging mouse model

Male C57BL/6J mice at different life stages, elderly (18 months) and juvenile (8 weeks), were obtained from Vital River Laboratory Animal Technology Co., Ltd., located in Beijing, China. These animals were maintained in a sterile environment meeting specific-pathogen-free standards within Central South University’s animal research center. Throughout the investigation, regulated parameters such as ambient temperature, moisture levels, and alternating 12-h periods of illumination and darkness were consistently upheld. Separately, to establish an aging model, 10-week-old mice received daily subcutaneous injections of D-gal (150 mg/kg; Sigma-Aldrich, USA) in the cervical and dorsal regions for 6 consecutive weeks. Tissue collection was performed at the end of this treatment period. The comparison group received equivalent quantities of physiological saline solution through identical administration methods.

The conditional *Pdha1* knockout mouse strain (C57BL/6J-*Pdha1*em1/V, VSM4106296) was developed through CRISPR/Cas9 genome editing technology by Beijing VIEWSOLID in China. The experimental procedure involved designing a circular donor plasmid containing *loxP* sequences inserted into the first intron. Researchers then performed cytoplasmic microinjection of Cas9 mRNA, single-guide RNAs, and the donor plasmid into fertilized single-cell embryos from C57BL/6J mice. This process produced chimeric founder animals that were later crossed with wild-type C57BL/6J mice to generate *Pdha1*^fl/+^ progeny with germline transmission. Successful targeting of the alleles was confirmed through Southern blot hybridization employing 2 distinct probes: a 3′ probe (following NcoI digestion; wild-type 4.1 kb, modified 2.8 kb) and a 5′ LR probe (after NdeI digestion).

The initial genetic modification involved a 5.7-kb wild-type sequence being replaced with a 2.8-kb targeted construct. Researchers subsequently bred the *Pdha*^fl/fl^ mouse line with another strain expressing Cre recombinase specifically in vascular ECs, driven by the natural Tek promoter/enhancer system (scientific designation: B6.Cg-Tg(*Tek*-CreERT2)/V mice, product code VSM30026, sourced from Beijing VIEWSOLID, China). This breeding strategy yielded mice with endothelial-specific deletion of the *Pdha1* gene (designated as *Pdha1*^fl/fl^/*Tek*^Cre^, abbreviated as *Pdha1*^ΔEC^ in the study documentation). To activate the Cre recombinase and induce endothelial-specific deletion of the *Pdha1* gene, 8-week-old male *Pdha1*^ΔEC^ and their control littermates received intraperitoneal injections of tamoxifen (75 mg/kg body weight, dissolved in corn oil) once daily for 5 consecutive days. Control animals received corn oil only. A standard washout period of 1 week was observed after the final injection to ensure complete clearance of tamoxifen and stabilization of gene recombination before any experimental procedures. The efficiency and specificity of *Pdha1* deletion in ECs were confirmed by genomic polymerase chain reaction (PCR) and immunofluorescence after this washout period. Throughout the investigation, animals carrying the Tek-Cre transgene but retaining intact *Pdha1* alleles (documented as *Pdha1*^WT^) served as the appropriate comparison group for the *Pdha1*^ΔEC^ experimental animals.

The *Mb21d1* gene is responsible for producing the cGAS protein, which plays a crucial role in triggering the TMEM173/STING signaling cascade to induce type I interferon synthesis. In these genetically modified animals, a targeted deletion was introduced in the *Mb21d1* locus (referred to as cGAS^KO^), specifically removing exon 2 that contains the enzymatic region. The creation of cGAS^KO^ mice involved a multistep breeding protocol. Initially, C57BL/6NTac-*Mb21d1*^tm1a(EUCOMM)Hmgu^/IcsOrl mice were mated with transgenic animals expressing FLPe recombinase under the control of the CAG promoter (C57BL/6-Tg (CAG-FLPe)36Ito/ltoRbrc) to eliminate the lacZ/neomycin selection marker, resulting in a conditional allele with flanked exon 2. These modified mice were then crossed with CMV-Cre transgenic lines (B6.C-Tg (CMV-cre)1Cgn/J) to achieve the final knockout. The genetically modified mice (Stock No. 006054) underwent targeted deletion of exon 2. These modified animals were subsequently bred with C57BL/6J mice to remove the Cre transgene. After arrival at our research center, further breeding with C57BL/6J mice (Stock No. 000664) was conducted to establish the colony. The cGAS-deficient mice were kindly supplied by Dr Ben Lu, having been initially acquired from The Jackson Laboratory (Catalog No. 026554).

### Ethical principles

The experimental protocols involving animals strictly adhered to the ethical guidelines set forth by Central South University’s Animal Care and Use Committee (located in Changsha, China) and were formally authorized by the Institutional Review Board of Xiangya Hospital of Central South University (Approval No. 2023030203). For the human component, lung tissue samples were obtained from patients aged over 60 years undergoing pulmonary resection. These patients were divided into a COPD group (forced expiratory volume in 1 s/forced vital capacity [FEV1/FVC] < 0.70), an idiopathic PF group (diagnosed according to the American Thoracic Society/European Respiratory Society criteria), and a control group (normal spirometry, neither diagnosis). Written consent forms were acquired from all human participants prior to sample collection. The research protocol was reviewed and approved by the designated ethics oversight panel (Approval No. 2022100983).

### PVEC isolation

PVECs were obtained from C57BL/6J mice through immunomagnetic separation employing the CD31 MicroBead Kit (130-097-418, Miltenyi Biotec, Germany). The experimental procedure commenced with administering sodium pentobarbital (100 mg/kg, intraperitoneal injection) to achieve profound anesthesia, followed by lung tissue extraction. The harvested tissues were subsequently flushed with heparin-containing phosphate-buffered saline (PBS) and mechanically dissected into smaller pieces. These tissue fragments were then subjected to enzymatic breakdown using 0.5% collagenase I (SCR103, Sigma, USA) at 37 °C for 50 min. The digested mixture was filtered to eliminate cellular debris and exposed to erythrocyte lysis solution (BL503B, Biosharp, China) under chilled conditions. After centrifugation, the cell pellet was resuspended in Dulbecco’s modified Eagle medium (DMEM), with total cell numbers quantified using an automated cell counting device (IC 1000, Countstar BioTech, China). For the magnetic separation process, aliquots containing 1 × 10^8^ cells were incubated with CD31-conjugated magnetic beads at 4 °C for 15 min. The labeled cellular components were then introduced into a specialized separation column placed within a magnetic field apparatus. Following thorough washing with a degassed buffer solution, the magnetic column was detached, and cells expressing CD31 were collected using pressure elution. Subsequently, the isolated PVECs were cultured in EC growth medium (1001, ScienCell, USA).

### Cell culture and experimental procedures

HUVECs were obtained from the American Type Culture Collection (RRID: CVCL_9Q53). These cells were incubated at 37 °C in a humidified 5% CO_2_ environment using DMEM (Gibco, Carlsbad, CA) enriched with 10% fetal bovine serum (Gibco) and 1% penicillin–streptomycin (Gibco). To investigate the influence of PDHA1 function in aging ECs, we pretreated the cells with either a PDHA1 inhibitor (3-FP, 15 μM, MedChemExpress, USA) or L-lactate (10 mM, MedChemExpress) 30 min prior to exposure to D-gal (30 mg/ml, Sigma-Aldrich). For PDHA1 activation, cells were incubated with the PDK inhibitor AZD7545 (40 μM, MedChemExpress) for 24 h.

### Plasmid construction and transfection

The expression vectors used in this study were procured from Sangon Biotech, a commercial supplier based in Shanghai, China. To create the PDHA1 variants S293A and K336R, site-specific mutagenesis was conducted. PCR amplification was carried out for both the native and modified gene sequences, employing specifically designed primers that contained NheI and HindIII recognition sequences at their 5′ and 3′ ends, respectively. Following purification, the amplified DNA fragments and pcDNA3.1 plasmid were cleaved simultaneously using the appropriate restriction enzymes. After purification of the digestion products, the target sequences were inserted into the prepared vector using T4 DNA ligase to generate the desired recombinant plasmids. Additionally, the PDHA1-specific short hairpin RNA (shRNA) lentiviral vector and its corresponding control plasmid (PDHA1 mouse shRNA Plasmid Kit) were acquired from Genechem Technologies.

In the transient gene transfer procedure, Lipofectamine 3000 (Invitrogen) was employed following the supplier’s recommended guidelines. Before the transfection process, both the plasmid DNA and Lipofectamine 3000 solution were individually prepared in a medium devoid of serum components. These solutions were subsequently mixed in optimal proportions and left undisturbed at ambient temperature for approximately 20 min to facilitate the formation of DNA–lipid complexes. The prepared transfection solution was carefully administered to the cell culture using a drop-by-drop method. After ensuring proper dispersion through gentle mixing, the original medium was exchanged with fresh complete medium supplemented with serum following an incubation period of 4 to 6 h. The transfected cells were maintained in culture conditions for another 48-h period prior to conducting subsequent analytical procedures or functional evaluations.

### Targeted metabolomics analysis

The experimental procedure involved plating cells into 6-cm culture dishes and permitting them to attach. After attachment was confirmed, the growth medium was removed, and the cellular monolayer underwent 2 PBS rinses. Subsequently, adherent cells were mechanically dislodged using a scraping tool, given an additional PBS wash, and quantified. From each experimental group (with 4 biological replicates), aliquots containing 1 × 10^7^ cells were concentrated by spinning at 2,500 rpm for 5 min under refrigerated conditions (4 °C). Following supernatant removal, the cellular precipitates were rapidly frozen using liquid nitrogen cryogenics and maintained at −80 °C for later metabolic profiling. Comprehensive metabolite profiling was performed by Shanghai Bioprofile (Shanghai, China). The extraction process commenced with the addition of ice-cold 80% methanol solution to each cellular sample, followed by thorough mixing to achieve complete cellular lysis. After a 5-min incubation on ice, the mixtures were subjected to high-speed centrifugation (15,000 × g, 15 min, 4 °C). The resulting liquid phase, containing the extracted metabolites, was carefully collected for subsequent analysis.

The samples were subjected to analytical procedures utilizing a Shimadzu Nexera LC-30AD ultrahigh-performance liquid chromatography (UHPLC) system (Shimadzu, Japan) connected to an AB SCIEX QTRAP 5500 mass spectrometric detector (AB Sciex, Framingham, MA, USA). The acquired UHPLC–tandem mass spectrometry data files were analyzed through the Compound Discoverer 3.1 software (Thermo Fisher Scientific) to perform peak alignment, feature extraction, and metabolite quantification. Metabolite identification was achieved by cross-referencing with the KEGG, Human Metabolome Database, and LIPID MAPS databases. Further statistical evaluation and metabolic pathway investigation were executed using the MetaboAnalyst 5.0 platform, with metabolic pathways showing marked enrichment being determined based on a *P*-value cutoff of less than 0.05.

### Lactate quantification

Lactate levels were measured after precise cell counting to ensure consistent cell numbers across all groups, as required by the Sigma lactate assay kit (which recommends harvesting 3 × 10^6^ to 5 × 10^6^ cells per assay). For each test, a combination of reagents was prepared, including 2 μl of the lactate detection reagent, 26 μl of the experimental sample, 26 μl of specialized buffer solution designed for lactate analysis, and 2 μl of enzymatic preparation. The samples were then allowed to react at ambient conditions for half an hour before analysis. Detection was performed through either colorimetric assessment at a 570-nm wavelength or fluorescence measurement using specific excitation (535 nm) and emission (587 nm) wavelengths. Lactate content was normalized to cell number and expressed as amount per 1 × 10^6^ cells.

### Western blot

For Western blot analysis, lung tissue and EC protein samples were prepared following established protocols from our prior research [69]. Tissue specimens and EC cultures were mechanically disrupted in radioimmunoprecipitation assay buffer (Solarbio, Beijing, China) supplemented with protease and phosphatase inhibitor mixtures. Protein quantification was performed through the bicinchoninic acid method. Aliquots containing 30 μg of protein or complete precipitated supernatant proteins were separated on 12% sodium dodecyl sulfate–polyacrylamide gel electrophoresis (SDS-PAGE) gels before being electro transferred onto polyvinylidene fluoride membranes (Millipore, USA). The membranes underwent blocking with either 5% skim milk or bovine serum albumin (BSA) solution for 90 min, followed by overnight incubation with primary antibodies at 4 °C. Subsequently, horseradish peroxidase-labeled secondary antibodies were applied for 60 min at ambient temperature. Protein bands were detected and analyzed using the Image Lab Analyzer software (Bio-Rad). The antibodies used in this study are summarized in Table 1.

**Table 1.** Antibody sources and dilutions

Antibodies	Source	Catalog	Dilution ratio
Primary antibodies for Western blot			
Rabbit anti-p16 polyclonal antibody	Abcam	Ab211542	1:2,000
Rabbit-anti-p21 polyclonal antibody	ABclonal	A22460	1:2,000
Rabbit anti-p53 polyclonal antibody	Proteintech	10442-1-AP	1:2,000
Anti-γ-H2AX polyclonal antibody	Boster	BM4841	1:2,000
Anti-HK2 monoclonal antibody	CST	#2867	1:2,000
Anti-LDHA-monoclonal antibody	Abcam	Ab52488	1:10,000
Anti-total OXPHOS complex antibody	Abcam	Ab110413	1:1,000
Anti-PDHA1 polyclonal antibody	Thermo Fisher	9H9AF5	1:2,000
Anti-p-PDHA1^Ser293^ monoclonal antibody	Thermo Fisher	MA535866	1:2,000
Anti-p-PDHA1^Ser300^ polyclonal antibody	Proteintech	29583-1-AP	1:2,000
Anti-p-PDHA1^Ser232^ monoclonal antibody	Proteintech	81491-1-RR	1:2,000
Anti-PDK1 polyclonal antibody	Proteintech	18262-1-AP	1:2,000
Anti-cGAS monoclonal antibody	CST	#31659	1: 1,000
Rabbit-anti-IRF3 antibody	Proteintech	11312-1-AP	1:10,000
Rabbit anti-p-IRF3^S396^ antibody	ABclonal	AP0623	1: 1,000
Anti-STING polyclonal antibody	Proteintech	19851-1-AP	1: 2,000
Anti-TBK1 polyclonal antibody	Proteintech	28397-1-AP	1: 2,000
Anti-p-TBK1 (Ser 172) polyclonal antibody	ABclonal	AP1026	1: 2,000
Anti-lactyl-lysine monoclonal antibody	PTM BioLab	PTM1401RM	1: 500
Rabbit-anti-Ki67 polyclonal antibody	Proteintech	27309-1-AP	1:100
Anti-α-tubulin monoclonal antibody	Servicebio	GB11200	1:10,000
Primary antibodies for IF			
Anti-p-PDHA1^Ser293^ polyclonal antibody	Thermo Fisher	MA535866	1:100
Anti-ZO-1 monoclonal antibody	Proteintech	21773-1-AP	1:100
Anti-CD31 polyclonal antibody	CST	3528	1:100
Rabbit-anti-CD31 polyclonal antibody	Proteintech	28083-1-AP	1:400
Anti-F4/80 polyclonal antibody	HUABIO	RT1212	1:100
Anti-SFTPC polyclonal antibody	Invitrogen	PA5-102493	1:1,000
Rabbit-anti-p21 polyclonal antibody	ABclonal	A1835	1:100
Rabbit-anti-p21 monoclonal antibody	CST	#2947	1:500
Rabbit-anti-p21 monoclonal antibody	Abcam	Ab18824	1:500
Secondary antibody			
HRP-conjugated goat anti-rabbit IgG	SAB	L3012-2	1: 5,000
Goat anti-mouse IgG	SAB	L3032	1: 5,000
Rabbit anti-goat IgG	SAB	L3042-2	1: 5,000
FITC-conjugated goat anti-rabbit IgG (H+L)	ABclonal	AS011	1:400
TRITC-conjugated goat anti-mouse IgG	ABclonal	AS026	1:400

γ-H2AX, phosphorylated histone H2AX; LDHA, lactate dehydrogenase A; HK2, hexokinase 2; IRF3, interferon regulatory factor 3; OXPHOS, oxidative phosphorylation; PDHA1, pyruvate dehydrogenase E1 component subunit alpha; PDK1, pyruvate dehydrogenase kinase 1; cGAS, cyclic GMP-AMP synthase; STING, stimulator of interferon genes; SIRT3, sirtuin 3; SFTPC, surfactant protein C; TBK1, TANK-binding kinase 1; IF, immunofluorescence staining; ZO-1, zonula occludens; HRP, horseradish peroxidase; IgG, immunoglobulin G; FITC, fluorescein isothiocyanate; TRITC, tetramethylrhodamine isothiocyanate

### Energy metabolism studies were conducted on ECs while maintaining their structural integrity

The metabolic activity of ECs was assessed using Seahorse XF24 Analyzer with the XF Cell Mito Stress Test Kit (Agilent, #103015-100). ECs were plated in Seahorse XF24 microplates at 10,000 cells per well and cultured overnight. One hour before the assay, the culture medium was replaced with 500 μl of assay medium (Seahorse XF DMEM [Agilent, #103575-100] supplemented with 10 mM glucose, 1 mM sodium pyruvate, and 2 mM L-glutamine, pH 7.4), followed by 60-min equilibration at 37 °C in a non-CO_2_ incubator. After calibration, a mitochondrial stress test was performed via WAVE Software (v2.6.1) with sequential injection of oligomycin (1.5 μM, port A), carbonyl cyanide *p*-trifluoromethoxyphenylhydrazone (FCCP; 1.0 μM, port B), and rotenone (0.5 μM, port C).

### SA-β-gal staining

The detection of SA-β-gal activity was carried out with a commercially available assay kit (Senescent Cell Histochemical Staining Kit, Beyotime Biotechnology, China) in strict accordance with the provided instructions. Briefly, following PBS washing, cellular specimens were subjected to fixation for 15 min under ambient conditions. The samples were then treated with the prepared staining solution and maintained at 37 °C for 24 h in an atmosphere devoid of carbon dioxide. Finally, the stained cellular components were examined using an inverted light microscope. The senescence rate was quantified by counting SA-β-gal-positive and total cells in 3 random fields per group, calculating the average percentage of positive cells. This value was then normalized to the control group (set as 1.0) and expressed as a fold change.

### Quantitative real-time PCR

The extraction of total RNA from both tissue samples and cell cultures was performed using the RNAiso Plus reagent (Takara Bio, Japan) in strict accordance with the supplier’s protocol. Spectrophotometric analysis was employed to evaluate RNA quality, with samples demonstrating an *A*_260_/*A*_280_ absorbance ratio above 1.8 being considered suitable for further processing. Reverse transcription was conducted with the PrimeScript RT reagent kit (Takara Bio), utilizing oligo(dT) primers to generate complementary DNA from the purified RNA. Gene expression analysis was performed through quantitative PCR using SYBR Green Master Mix (Applied Biosystems) on a CFX96 Touch instrument (Bio-Rad, USA) at Central South University. *β-actin* served as the reference gene for normalization, and relative gene expression levels were calculated using the comparative threshold cycle (2^−ΔΔCt^) approach. All amplification primers were designed through NCBI Primer-BLAST, with their specific nucleotide sequences detailed in Table 2.

**Table 2.** Sequences of the primers used to quantify gene expression

Gene	Forward primer (5′–3′)	Reverse primer (5′–3′)
*m-p16*	CTCTGCTCTTGGGATTGGC	GTGCGATATTTGCGTTCCG
*m-p19*	GAGGCCGGCAAATGATCATAGA	GTGGATACCGGTGGACTGTG
*m-p21*	GTGAGGAGGAGCATGAATGGA	GAACAGGTCGGACATCACCA
*m-p53*	ATGACTGCCATGGAGGAGTCAC	TCAGTCTGAGTCAGGCCCCAC
*m-Il-1β*	CAGGCAGGCAGTATCACTCA	AGCTCATATGGGTCCGACAG
*m-Il-18*	ACGTGTTCCAGGACACAACA	CAAACCCTCCCCACCTAACT
*m-D-loop1*	CCCTTCCCCATTTGGTCT	TGGTTTCACGGAGGATGG
*m-Cytb*	GCTTTCCACTTCATCTTACCATTTA	TGTTGGGTTGTTTGATCCTG
*m-Nd-1*	TATCTCAACCCTAGCAGAAA	TAACGCGAATGGGCCGGCTG
*m-Pdha1*	TGTGACCTTCATCGGCTAGAA	TGATCCGCCTTTAGCTCCATC
*m-Cre^ERT^*	CGCTAAGGATGACTCTGG	CAACAAGGCACTGACCAT
*β-actin*	TTCCAGCCTTCCTTCTTG	GGAGCCAGAGCAGTAATC

### Flow cytometry

To prepare samples for flow cytometry analysis, primary ECs from mice were first dissociated into individual cell units. The collected cells were then placed in chilled PBS and treated with a fluorescein isothiocyanate-labeled CD31-specific antibody (Proteintech, FITC-65058). After thorough rinsing with PBS, the cellular suspension was transferred into a specialized buffer solution designed for flow cytometry applications. The prepared samples were subsequently examined using a BD FACS Verse instrument manufactured by BD Biosciences. The resulting data were processed and interpreted through the FlowJo analytical software (version 10, Tree Star Inc., USA).

### Evaluation of the PDHC enzymatic function

The enzymatic activity of PDHC was quantified utilizing the commercially available PDHC Activity Assay Kit (Solarbio, BC0385). This assay relies on the principle that PDH facilitates the oxidative decarboxylation of pyruvate while simultaneously causing the reduction of 2,6-dichlorophenolindophenol, resulting in diminished light absorption at a wavelength of 605 nm. Optical density measurements were taken at this specific wavelength at both the initial time point (0 min, designated as *A*_1_) and after 60 s (1 min, designated as *A*_2_) using a microplate reader designed for enzyme-linked immunosorbent assay applications. The differential in absorbance values (Δ*A* = *A*_1_ − *A*_2_) was subsequently employed to determine PDHC enzymatic activity following the standardized procedures provided by the kit manufacturer.

### Morphometric analysis

Left pulmonary lobes were excised post-euthanasia, fixed in 4% neutral-buffered formaldehyde at 4 °C and processed for paraffin embedding. Sections (4 μm) were stained with hematoxylin and eosin (Solarbio), and whole-slide images were captured using a Pannoramic Scan system (3Dhistech, Budapest, Hungary). The quantitative evaluation of the mean linear intercept and destructive index was performed according to established protocols [70]. The mean linear intercept was derived from the ratio of total linear length to septal intersection counts, and the destructive index was calculated as the proportion of disrupted alveoli relative to the total alveolar number.

### Immunofluorescence staining

Immunofluorescence analysis was carried out on lung tissue sections with a thickness of 3 μm embedded in paraffin or on cell cultures postfixation. After rinsing with PBS, the specimens were treated with 4% paraformaldehyde solution for 15 min under ambient conditions. For lung tissue sections, antigen retrieval was performed by high-pressure treatment in sodium citrate buffer (10 mM, pH 6.0) for 2 min, followed by cooling to room temperature. Cell membrane permeability was enhanced by applying 0.1% Tween-20 detergent solution. To minimize background staining, a blocking step was performed using 10% BSA in PBS buffer for 60 min at room temperature. Primary antibody incubation proceeded for approximately 16 h at 4 °C. Subsequent to thorough PBS rinses, fluorescent dye-conjugated secondary antibodies corresponding to the host species were introduced and allowed to react for 60 min under light-protected conditions at ambient temperature. Nuclear counterstaining was achieved with 4′,6-diamidino-2-phenylindole from Solarbio. Fluorescence images were acquired using a Leica SP8 confocal laser scanning microscope manufactured in Germany.

### Y-maze test

The evaluation of spatial working memory and novelty-seeking behavior was conducted through a Y-maze experiment, adhering to standardized methodology [56]. The experimental setup featured a 3-branched wooden maze, with distinct arms labeled as the initial, known, and unfamiliar sections. The assessment protocol involved 2 distinct stages: habituation and evaluation. In the habituation stage, access to the unfamiliar arm was restricted, permitting rodents to investigate only the initial and known arms for a duration of 5 min. Following a 120-min rest period, the evaluation stage began with all maze arms accessible. Subjects were placed back in the initial arm, and their movement patterns across all 3 arms were monitored for 5 min using the Smart 3.0 automated tracking system. Key measurements included the duration and frequency of visits to the unfamiliar arm, along with entry patterns to determine spontaneous alternation rates, which serve as indicators of spatial working memory capacity.

### Open-field test

Motor and exploratory behaviors were assessed through the OFT. Each rodent was positioned at the midpoint of a square testing chamber measuring 100 × 100 × 40 cm and permitted to move without restriction for a 5-min period. Researchers documented and analyzed the duration that each subject remained in the central area. To prevent scent interference, the testing area was meticulously sanitized with 75% ethanol solution and completely dried before subsequent trials.

### Elevated plus maze

Anxiety-like responses were examined using the EPM apparatus, which consisted of 2 exposed and 2 enclosed arms extending from a central platform, elevated 50 cm above the ground. Test subjects were introduced to the central zone and given 5 min to navigate the maze. The level of anxiety reduction was determined by calculating both the time allocated to and the number of visits to the unprotected arms.

### Mitochondrial reactive oxygen species

mtROS were detected using MitoSOX Red reagent (Thermo Fisher Scientific, M36009). A stock solution was prepared by diluting the reagent 1:1,000 in serum-free medium to a final concentration of 1 μM. After experimental treatments, cells cultured in 24-well plates or glass-bottom dishes were washed 3 times with prewarmed 1× PBS (5 min per wash) under gentle agitation on an orbital shaker (37 °C, 50 rpm). The cells were then incubated with 500 μl of the staining solution per well for 30 min at 37 °C in the dark. After incubation, the staining solution was removed, and the cells were washed 3 times with PBS. Nuclei were counterstained with Hoechst 33342 (10 μg/ml, Solarbio, C0030) for 10 min at 37 °C in the dark, followed by 3 additional PBS washes. Samples were immediately examined by fluorescence microscopy.

### Measurement of mitochondrial membrane potential

The assessment of mitochondrial membrane potential was conducted with the JC-1 Assay Kit (Beyotime) in strict accordance with the provided protocol. PVEC samples underwent PBS washing before being treated with JC-1 staining mixture, which was formulated by combining equal parts of RPMI 1640 medium and JC-1 working solution. This incubation process occurred at 37 °C for a duration of 20 min under light-protected conditions. Following staining, the samples were analyzed by both fluorescence microscopy and flow cytometry. Fluorescent microscopy was employed to capture images, followed by computational analysis using the ImageJ software to determine the proportional relationship between red and green fluorescence intensities. Flow cytometric analysis was performed on a flow cytometer, and the data were analyzed to assess the shift in red/green fluorescence ratio, indicating changes in mitochondrial membrane potential.

### Analysis of cytoplasmic mtDNA

The procedure for isolating and measuring cytosolic mtDNA followed established protocols [71]. In detail, PVECs were plated into 6-well culture dishes at a concentration of 1 million cells per well. Upon achieving full confluence, the cell membranes were disrupted using 1% NP-40 detergent while maintaining samples on ice for 20 min. Subsequent centrifugation at 16,000 × g for 15 min at 4 °C separated cellular fragments from the soluble fraction. The QIAamp DNA Mini Kit (Qiagen, Germany) was employed to purify genomic DNA from the obtained cytosolic fraction. Quantitative assessment of mtDNA content was performed through real-time PCR, utilizing specific primer sets designed for mitochondrial genomic segments including cytochrome b (*Cytb*), NADH dehydrogenase 1 (*Nd1*), and the displacement loop region.

### Co-IP assay

To examine interactions between proteins, researchers performed a Co-IP experiment utilizing the Universal IP/Co-IP Kit (Agarose, KTD105-CN, Abbkine), following the supplier’s instructions. Cell lysates were obtained from 5 million cells by treating them with 1 ml of Non-Denaturing Lysis Buffer (supplied with the Universal IP/Co-IP Kit, Abbkine, KTD105-CN; used according to the manufacturer’s protocol) supplemented with protease inhibitors (MedChemExpress, HY-K0010) for 15 min on ice. The mixture was then centrifuged at 12,000 × g for 10 min at 4 °C to collect the supernatant. Concurrently, the immunoprecipitation matrix was prepared by rinsing 40 μl of Protein A/G Agarose beads with wash buffer and incubating them with 1 μg of anti-PDHA1 antibody for 1 h at ambient temperature. Subsequently, 1 mg of the protein sample was mixed with the antibody-bound beads and left to incubate at 4 °C overnight. Following this, the immunocomplexes were separated by centrifugation, washed thoroughly, and heated in 50 μl of SDS-PAGE loading buffer at 95 °C for 10 min. The resulting protein samples were then subjected to Western blot analysis for further evaluation.

### Statistical analysis

Each experimental procedure was replicated a minimum of 3 separate occasions. All data follow a normal distribution, which was tested with the Shapiro–Wilk test. Results are displayed as averages with standard deviations. The GraphPad Prism 9 software (San Diego, CA, USA) was employed for all statistical evaluations. A significance threshold of *P* < 0.05 was established for determining statistical relevance. For pairwise comparisons, an unpaired Student *t* test was utilized, while multiple group analyses were conducted using one-way analysis of variance with subsequent Tukey’s or Dunnett’s post hoc testing.

## Data Availability

All data are available in the main text or the Supplementary Materials.

## References

[1] Grunewald M, Kumar S, Sharife H, Volinsky E, Gileles-Hillel A, Licht T, Permyakova A, Hinden L, Azar S, Friedmann Y, et al. Counteracting age-related VEGF signaling insufficiency promotes healthy aging and extends life span. Science. 2021;373(6554): Article eabc8479. 34326210 10.1126/science.abc8479

[2] Schuliga M, Read J, Knight DA. Ageing mechanisms that contribute to tissue remodeling in lung disease. Ageing Res Rev. 2021;70: Article 101405. 34242806 10.1016/j.arr.2021.101405

[3] Raslan AA, Pham TX, Lee J, Kontodimas K, Tilston-Lunel A, Schmottlach J, Hong J, Dinc T, Bujor AM, Caporarello N, et al. Lung injury-induced activated endothelial cell states persist in aging-associated progressive fibrosis. Nat Commun. 2024;15(1):5449. 38937456 10.1038/s41467-024-49545-x PMC11211333

[4] Ackermann M, Werlein C, Plucinski E, Leypold S, Kühnel MP, Verleden SE, Khalil HA, Länger F, Welte T, Mentzer SJ, et al. The role of vasculature and angiogenesis in respiratory diseases. Angiogenesis. 2024;27(3):293–310. 38580869 10.1007/s10456-024-09910-2 PMC11303512

[5] Bloom SI, Islam MT, Lesniewski LA, Donato AJ. Mechanisms and consequences of endothelial cell senescence. Nat Rev Cardiol. 2023;20(1):38–51. 35853997 10.1038/s41569-022-00739-0 PMC10026597

[6] Du Y, Zhu P, Li Y, Yu J, Xia T, Chang X, Zhu H, Li R, He Q. DNA-PKcs phosphorylates cofilin2 to induce endothelial dysfunction and microcirculatory disorder in endotoxemic cardiomyopathy. Research. 2024;7:0331. 38550779 10.34133/research.0331 PMC10976589

[7] Jin C, Zhang DP, Lin Z, Lin YZ, Shi YF, Dong XY, Jin MQ, Song FQ, Du ST, Feng YZ, et al. Piezo1-mediated ferroptosis delays wound healing in aging mice by regulating the transcriptional activity of SLC7A11 through activating transcription factor 3. Research. 2025;8:0718. 40463502 10.34133/research.0718 PMC12133029

[8] Zhang H, Liu S, Fu S, Zhao Q, Wang Y, Yuan Y, Zhang C. Novel insight into the Warburg effect: Sweet temptation. Crit Rev Oncol Hematol. 2025;214: Article 104844. 40639495 10.1016/j.critrevonc.2025.104844

[9] Zhang F, Guo J, Yu S, Zheng Y, Duan M, Zhao L, Wang Y, Yang Z, Jiang X. Cellular senescence and metabolic reprogramming: Unraveling the intricate crosstalk in the immunosuppressive tumor microenvironment. Cancer Commun. 2024;44(9):929–966. 10.1002/cac2.12591 PMC11492308 38997794

[10] Zhong WJ, Liu T, Yang HH, Duan JX, Yang JT, Guan XX, Xiong JB, Zhang YF, Zhang CY, Zhou Y, et al. TREM-1 governs NLRP3 inflammasome activation of macrophages by firing up glycolysis in acute lung injury. Int J Biol Sci. 2023;19(1):242–257. 36594089 10.7150/ijbs.77304 PMC9760435

[11] Lu C, Gao R, Qing P, Zeng X, Liao X, Cheng M, Qin L, Liu Y. Single-cell transcriptome analyses reveal disturbed decidual homoeostasis in obstetric antiphospholipid syndrome. Ann Rheum Dis. 2024;83(5):624–637. 38331588 10.1136/ard-2023-224930

[12] Lian J, Yue Y, Yu W, Zhang Y. Immunosenescence: A key player in cancer development. J Hematol Oncol. 2020;13(1):151. 33168037 10.1186/s13045-020-00986-z PMC7653700

[13] De Bock K, Georgiadou M, Schoors S, Kuchnio A, Wong BW, Cantelmo AR, Quaegebeur A, Ghesquiere B, Cauwenberghs S, Eelen G, et al. Role of PFKFB3-driven glycolysis in vessel sprouting. Cell. 2013;154(3):651–663. 23911327 10.1016/j.cell.2013.06.037

[14] Li X, Sun X, Carmeliet P. Hallmarks of endothelial cell metabolism in health and disease. Cell Metab. 2019;30(3):414–433. 31484054 10.1016/j.cmet.2019.08.011

[15] Wu Y, Tang L, Huang H, Yu Q, Hu B, Wang G, Ge F, Yin T, Li S, Yu X. Phosphoglycerate dehydrogenase activates PKM2 to phosphorylate histone H3T11 and attenuate cellular senescence. Nat Commun. 2023;14(1):1323. 36899022 10.1038/s41467-023-37094-8 PMC10006232

[16] Yan K, He Q, Lin D, Liang J, Chen J, Xie Z, Chen Z. Promotion of NAD^+^ recycling by the hypoxia-induced shift in the lactate dehydrogenase isozyme profile reduces the senescence of human bone marrow-derived endothelial progenitor cells. Free Radic Biol Med. 2023;208:88–102. 37536460 10.1016/j.freeradbiomed.2023.07.035

[17] Duarte IF, Caio J, Moedas MF, Rodrigues LA, Leandro AP, Rivera IA, Silva MF. Dihydrolipoamide dehydrogenase, pyruvate oxidation, and acetylation-dependent mechanisms intersecting drug iatrogenesis. Cell Mol Life Sci. 2021;78(23):7451–7468. 34718827 10.1007/s00018-021-03996-3 PMC11072406

[18] Zhou ZH, McCarthy DB, O’Connor CM, Reed LJ, Stoops JK. The remarkable structural and functional organization of the eukaryotic pyruvate dehydrogenase complexes. Proc Natl Acad Sci USA. 2001;98(26):14802–14807. 11752427 10.1073/pnas.011597698 PMC64939

[19] Patel MS, Nemeria NS, Furey W, Jordan F. The pyruvate dehydrogenase complexes: Structure-based function and regulation. J Biol Chem. 2014;289(24):16615–16623. 24798336 10.1074/jbc.R114.563148 PMC4059105

[20] Hitosugi T, Fan J, Chung TW, Lythgoe K, Wang X, Xie J, Ge Q, Gu TL, Polakiewicz RD, Roesel JL, et al. Tyrosine phosphorylation of mitochondrial pyruvate dehydrogenase kinase 1 is important for cancer metabolism. Mol Cell. 2011;44(6):864–877. 22195962 10.1016/j.molcel.2011.10.015 PMC3246218

[21] Cai Z, Li CF, Han F, Liu C, Zhang A, Hsu CC, Peng D, Zhang X, Jin G, Rezaeian AH, et al. Phosphorylation of PDHA by AMPK drives TCA cycle to promote cancer metastasis. Mol Cell. 2020;80(2):263–278 e267. 33022274 10.1016/j.molcel.2020.09.018 PMC7534735

[22] Cevatemre B, Ulukaya E, Dere E, Dilege S, Acilan C. Pyruvate dehydrogenase contributes to drug resistance of lung cancer cells through epithelial mesenchymal transition. Front Cell Dev Biol. 2021;9: Article 738916. 35083212 10.3389/fcell.2021.738916 PMC8785343

[23] Zhang N, Sun L, Zhou S, Ji C, Cui T, Chu Q, Ye J, Liang S, Ma K, Liu Y, et al. Cholangiocarcinoma PDHA1 succinylation suppresses macrophage antigen presentation via alpha-ketoglutaric acid accumulation. Nat Commun. 2025;16(1):3177. 40180922 10.1038/s41467-025-58429-7 PMC11968997

[24] Tran TTV, Jeong Y, Kim S, Yeom JE, Lee J, Lee W, Bae GU, Kang JS. PRMT1 ablation in endothelial cells causes endothelial dysfunction and aggravates COPD attributable to dysregulated NF-κB signaling. Adv Sci. 2025;12(19): Article e2411514. 10.1002/advs.202411514 PMC12097043 40135804

[25] Mobus L, Yla-Outinen L, Mannino L, Migliaccio G, Kosunen K, D’Alessandro N, Serra A, Greco D. Endothelial sensitivity to pro-fibrotic signals links systemic exposure to pulmonary fibrosis. Cell Death Dis. 2025;16(1):500. 40623988 10.1038/s41419-025-07824-5 PMC12234957

[26] Locker F, Bieler L, Nowack LMF, Leitner J, Brunner SM, Zaunmair P, Kofler B, Couillard-Despres S. Involvement of neuropeptide galanin receptors 2 and 3 in learning, memory and anxiety in aging mice. Molecules. 2021;26(7):1978. 33915732 10.3390/molecules26071978 PMC8037218

[27] Chen J, Guccini I, Di Mitri D, Brina D, Revandkar A, Sarti M, Pasquini E, Alajati A, Pinton S, Losa M, et al. Compartmentalized activities of the pyruvate dehydrogenase complex sustain lipogenesis in prostate cancer. Nat Genet. 2018;50(2):219–228. 29335542 10.1038/s41588-017-0026-3 PMC5810912

[28] Jin L, Cho M, Kim BS, Han JH, Park S, Lee IK, Ryu D, Kim JH, Bae SJ, Ha KT. Drug evaluation based on phosphomimetic PDHA1 reveals the complexity of activity-related cell death in A549 non-small cell lung cancer cells. BMB Rep. 2021;54(11):563–568. 34488935 10.5483/BMBRep.2021.54.11.101 PMC8633525

[29] Fan J, Shan C, Kang HB, Elf S, Xie J, Tucker M, Gu TL, Aguiar M, Lonning S, Chen H, et al. Tyr phosphorylation of PDP1 toggles recruitment between ACAT1 and SIRT3 to regulate the pyruvate dehydrogenase complex. Mol Cell. 2014;53(4):534–548. 24486017 10.1016/j.molcel.2013.12.026 PMC3943932

[30] Mao Y, Zhang J, Zhou Q, He X, Zheng Z, Wei Y, Zhou K, Lin Y, Yu H, Zhang H, et al. Hypoxia induces mitochondrial protein lactylation to limit oxidative phosphorylation. Cell Res. 2024;34(1):13–30. 38163844 10.1038/s41422-023-00864-6 PMC10770133

[31] Inigo JR, Chandra D. The mitochondrial unfolded protein response (UPR^mt^): Shielding against toxicity to mitochondria in cancer. J Hematol Oncol. 2022;15(1):98. 35864539 10.1186/s13045-022-01317-0 PMC9306209

[32] Zhong WJ, Yang HH, Guan XX, Xiong JB, Sun CC, Zhang CY, Luo XQ, Zhang YF, Zhang J, Duan JX, et al. Inhibition of glycolysis alleviates lipopolysaccharide-induced acute lung injury in a mouse model. J Cell Physiol. 2019;234(4):4641–4654. 30256406 10.1002/jcp.27261

[33] Penniman CM, Bhardwaj G, Nowers CJ, Brown CU, Junck TL, Boyer CK, Jena J, Fuqua JD, Lira VA, O’Neill BT. Loss of FoxOs in muscle increases strength and mitochondrial function during aging. J Cachexia Sarcopenia Muscle. 2023;14(1):243–259. 36442857 10.1002/jcsm.13124 PMC9891940

[34] Korotchkina LG, Patel MS. Mutagenesis studies of the phosphorylation sites of recombinant human pyruvate dehydrogenase. Site-specific regulation. J Biol Chem. 1995;270(24):14297–14304. 7782287 10.1074/jbc.270.24.14297

[35] Korotchkina LG, Patel MS. Site specificity of four pyruvate dehydrogenase kinase isoenzymes toward the three phosphorylation sites of human pyruvate dehydrogenase. J Biol Chem. 2001;276(40):37223–37229. 11486000 10.1074/jbc.M103069200

[36] Kato M, Wynn RM, Chuang JL, Tso SC, Machius M, Li J, Chuang DT. Structural basis for inactivation of the human pyruvate dehydrogenase complex by phosphorylation: Role of disordered phosphorylation loops. Structure. 2008;16(12):1849–1859. 19081061 10.1016/j.str.2008.10.010 PMC2849990

[37] Xin MG, Zhang J, Block ER, Patel JM. Senescence-enhanced oxidative stress is associated with deficiency of mitochondrial cytochrome *c* oxidase in vascular endothelial cells. Mech Ageing Dev. 2003;124(8–9):911–919. 14499496 10.1016/s0047-6374(03)00163-5

[38] Zhang J, Block ER, Patel JM. Down-regulation of mitochondrial cytochrome *c* oxidase in senescent porcine pulmonary artery endothelial cells. Mech Ageing Dev. 2002;123(10):1363–1374. 12297339 10.1016/s0047-6374(02)00075-1

[39] Kumazaki T, Sakano T, Yoshida T, Hamada K, Sumida H, Teranishi Y, Nishiyama M, Mitsui Y. Enhanced expression of mitochondrial genes in senescent endothelial cells and fibroblasts. Mech Ageing Dev. 1998;101(1–2):91–99. 9593315 10.1016/s0047-6374(97)00159-0

[40] Toto Nienguesso A, Jung JS, Alfes M, Schindler M, Täubert L, Schmidt C, Navarrete Santos A. Age-related changes in the proteome and mitochondrial metabolism of rabbit adipose-derived stromal/stem cells. Sci Rep. 2025;15(1):20183. 40542110 10.1038/s41598-025-06030-9 PMC12181353

[41] Phang HJ, Bergstrom J, Keri B, Heimler SR, Dozier S, Scandalis LM, Wing D, Moreno D, Sun NN, Molina AJ. Blood cell mitochondrial respiration increases with age and varies by sex in healthy adults. Aging Cell. 2026;25(2): Article e70387. 41566162 10.1111/acel.70387 PMC12823460

[42] Wang CH, Lu WL, Chiang SL, Tsai TH, Liu SC, Hsieh CH, Su PH, Huang CY, Tsai FJ, Lin YJ, et al. T cells mediate kidney tubular injury via impaired PDHA1 and autophagy in type 1 diabetes. J Clin Endocrinol Metab. 2022;107(9):2556–2570. 35731579 10.1210/clinem/dgac378

[43] Chen Z, Liu X, Zuo K, Xin Y, Liu J. TAT-PBX1 fusion protein alleviates LPS-induced acute lung injury via AMPK-TFAM signaling activation. Mol Ther. 2025;33(12):6537–6553. 40926413 10.1016/j.ymthe.2025.09.010 PMC12703146

[44] Zhuge A, Li S, Han S, Yuan Y, Shen J, Wu W, Wang K, Xia J, Wang Q, Gu Y, et al. *Akkermansia muciniphila*-derived acetate activates the hepatic AMPK/SIRT1/PGC-1*α* axis to alleviate ferroptosis in metabolic-associated fatty liver disease. Acta Pharm Sin B. 2025;15(1):151–167. 40041901 10.1016/j.apsb.2024.10.010 PMC11873632

[45] Villar-Vesga J, De Feo D, Clement P, Bugada V, Meuffels E, Van Hove H, Bijnen M, King J, Grundschober S, Ulutekin C, et al. Monocyte-derived macrophages drive neurological tissue damage through mitochondrial reactive oxygen species. Sci Immunol. 2026;11(119):eadw5197. 42102230 10.1126/sciimmunol.adw5197

[46] Jomova K, Raptova R, Alomar SY, Alwasel SH, Nepovimova E, Kuca K, Valko M. Reactive oxygen species, toxicity, oxidative stress, and antioxidants: Chronic diseases and aging. Arch Toxicol. 2023;97(10):2499–2574. 37597078 10.1007/s00204-023-03562-9 PMC10475008

[47] Zorov DB, Juhaszova M, Sollott SJ. Mitochondrial reactive oxygen species (ROS) and ROS-induced ROS release. Physiol Rev. 2014;94(3):909–950. 24987008 10.1152/physrev.00026.2013 PMC4101632

[48] Lee HC, Yin PH, Chi CW, Wei YH. Increase in mitochondrial mass in human fibroblasts under oxidative stress and during replicative cell senescence. J Biomed Sci. 2002;9(6 Pt 1):517–526. 12372989 10.1007/BF02254978

[49] Korolchuk VI, Miwa S, Carroll B, von Zglinicki T. Mitochondria in cell senescence: Is mitophagy the weakest link? EBioMedicine. 2017;21:7–13. 28330601 10.1016/j.ebiom.2017.03.020 PMC5514379

[50] Schoonjans CA, Mathieu B, Joudiou N, Zampieri LX, Brusa D, Sonveaux P, Feron O, Gallez B. Targeting endothelial cell metabolism by inhibition of pyruvate dehydrogenase kinase and glutaminase-1. J Clin Med. 2020;9(10):3308. 33076309 10.3390/jcm9103308 PMC7602423

[51] Zhang CY, Zhong WJ, Liu YB, Duan JX, Jiang N, Yang HH, Ma SC, Jin L, Hong JR, Zhou Y, et al. EETs alleviate alveolar epithelial cell senescence by inhibiting endoplasmic reticulum stress through the Trim25/Keap1/Nrf2 axis. Redox Biol. 2023;63: Article 102765. 37269686 10.1016/j.redox.2023.102765 PMC10249012

[52] Zhong WJ, Xiong JB, Zhang CY, Jin L, Yang NS, Sha HX, Liu YB, Duan JX, Guan CX, Zhou Y, et al. Blocking triggering receptors expressed on myeloid cell-1 alleviates alveolar epithelial cell senescence by inhibiting oxidative stress in pulmonary fibrosis. Histochem Cell Biol. 2025;163(1):45. 40240638 10.1007/s00418-025-02374-5

[53] Zhong WJ, Zhang CY, Duan JX, Chen MR, Zhang BL, Yang NS, Sha HX, Zhang J, Xiong JB, Guan CX, et al. Krüppel-like transcription factor 14 alleviates alveolar epithelial cell senescence by inhibiting endoplasmic reticulum stress in pulmonaryfibrosis. Int J Biol Macromol. 2024;280(Pt 1) Article 135351. 39270890 10.1016/j.ijbiomac.2024.135351

[54] Kaplon J, Zheng L, Meissl K, Chaneton B, Selivanov VA, Mackay G, Van Der Burg SH, Verdegaal EM, Cascante M, Shlomi T, et al. A key role for mitochondrial gatekeeper pyruvate dehydrogenase in oncogene-induced senescence. Nature. 2013;498(7452):109–112. 23685455 10.1038/nature12154

[55] Wiley CD, Campisi J. The metabolic roots of senescence: Mechanisms and opportunities for intervention. Nat Metab. 2021;3(10):1290–1301. 34663974 10.1038/s42255-021-00483-8 PMC8889622

[56] Zhong WJ, Sha HX, Zhang CY, Jin L, Duan JX, Xiong JB, You ZJ, Zhou Y, Guan CX. mtDNA-cGAS-STING axis-dependent NLRP3 inflammasome activation contributes to postoperative cognitive dysfunction induced by sevoflurane in mice. Int J Biol Sci. 2024;20(5):1927–1946. 38481801 10.7150/ijbs.91543 PMC10929193

[57] Sha HX, Liu YB, Qiu YL, Zhong WJ, Yang NS, Zhang CY, Duan JX, Xiong JB, Guan CX, Zhou Y. Neutrophil extracellular traps trigger alveolar epithelial cell necroptosis through the cGAS-STING pathway during acute lung injury in mice. Int J Biol Sci. 2024;20(12):4713–4730. 39309425 10.7150/ijbs.99456 PMC11414388

[58] Liu H, Ghosh S, Vaidya T, Bammidi S, Huang C, Shang P, Nair AP, Chowdhury O, Stepicheva NA, Strizhakova A, et al. Activated cGAS/STING signaling elicits endothelial cell senescence in early diabetic retinopathy. JCI Insight. 2023;8(12): Article e168945. 37345657 10.1172/jci.insight.168945 PMC10371250

[59] Yu H, Liao K, Hu Y, Lv D, Luo M, Liu Q, Huang L, Luo S. Role of the cGAS-STING pathway in aging-related endothelial dysfunction. Aging Dis. 2022;13(6):1901–1918. 36465181 10.14336/AD.2022.0316 PMC9662267

[60] Peng J, Zhang Q, Rao X, Allison DB, Kong Y, Wang R, Liu J, Zhang Y, Katz W, Li Z, et al. PLK1-mediated PDHA1 phosphorylation drives metabolic reprogramming in lung cancer. Oncogene. 2025;44(43):4190–4204. 40957950 10.1038/s41388-025-03571-1 PMC12503065

[61] Leutert M, Entwisle SW, Villen J. Decoding post-translational modification crosstalk with proteomics. Mol Cell Proteomics. 2021;20: Article 100129. 34339852 10.1016/j.mcpro.2021.100129 PMC8430371

[62] Liu Y, Feng W, Wang Y, Wu B. Crosstalk between protein post-translational modifications and phase separation. Cell Commun Signal. 2024;22(1):110. 38347544 10.1186/s12964-023-01380-1 PMC10860296

[63] Zhang D, Gao J, Zhu Z, Mao Q, Xu Z, Singh PK, Rimayi CC, Moreno-Yruela C, Xu S, Li G, et al. Lysine L-lactylation is the dominant lactylation isomer induced by glycolysis. Nat Chem Biol. 2025;21(1):91–99. 39030363 10.1038/s41589-024-01680-8 PMC11666458

[64] Jing E, O’Neill BT, Rardin MJ, Kleinridders A, Ilkeyeva OR, Ussar S, Bain JR, Lee KY, Verdin EM, Newgard CB, et al. Sirt3 regulates metabolic flexibility of skeletal muscle through reversible enzymatic deacetylation. Diabetes. 2013;62(10):3404–3417. 23835326 10.2337/db12-1650 PMC3781465

[65] Song Y, Wan X, Gao L, Pan Y, Xie W, Wang H, Guo J. Activated PKR inhibits pancreatic β-cell proliferation through sumoylation-dependent stabilization of P53. Mol Immunol. 2015;68(2 Pt A):341–349. 26446704 10.1016/j.molimm.2015.09.007

[66] Wu Z, Huang R, Yuan L. Crosstalk of intracellular post-translational modifications in cancer. Arch Biochem Biophys. 2019;676: Article 108138. 31606391 10.1016/j.abb.2019.108138

[67] Oh CJ, Kim MJ, Lee JM, Kim DH, Kim IY, Park S, Kim Y, Lee KB, Lee SH, Lim CW, et al. Inhibition of pyruvate dehydrogenase kinase 4 ameliorates kidney ischemia-reperfusion injury by reducing succinate accumulation during ischemia and preserving mitochondrial function during reperfusion. Kidney Int. 2023;104(4):724–739. 37399974 10.1016/j.kint.2023.06.022

[68] Zhao X, Yang X, Du C, Hao H, Liu S, Liu G, Zhang G, Fan K, Ma J. Up-regulated succinylation modifications induce a senescence phenotype in microglia by altering mitochondrial energy metabolism. J Neuroinflammation. 2024;21(1):296. 39543710 10.1186/s12974-024-03284-4 PMC11566524

[69] Yang HH, Jiang HL, Tao JH, Zhang CY, Xiong JB, Yang JT, Liu YB, Zhong WJ, Guan XX, Duan JX, et al. Mitochondrial citrate accumulation drives alveolar epithelial cell necroptosis in lipopolysaccharide-induced acute lung injury. Exp Mol Med. 2022;54(11):2077–2091. 36443565 10.1038/s12276-022-00889-8 PMC9722936

[70] Duan JX, Guan XX, Cheng W, Deng DD, Chen P, Liu C, Zhou Y, Hammock BD, Yang HH. COX-2/sEH-mediated macrophage activation is a target for pulmonary protection in mouse models of chronic obstructive pulmonary disease. Lab Investig. 2024;104(3): Article 100319. 38158123 10.1016/j.labinv.2023.100319

[71] Zhang CY, Ou AJ, Jin L, Yang NS, Deng P, Guan CX, Huang XT, Duan JX, Zhou Y. Cadmium exposure triggers alveolar epithelial cell pyroptosis by inducing mitochondrial oxidative stress and activating the cGAS-STING pathway. Cell Commun Signal. 2024;22(1):566. 39587603 10.1186/s12964-024-01946-7 PMC11590492

