# Supplementary material for: PDHA1 Hyperactivation Orchestrates Metabolic Reprogramming Promoting Endothelial Senescence
Source: Research (Wash D C). 2026 Aug 20;9:1398. doi: 10.34133/research.1398 (PMC13490744; doi:10.34133/research.1398)
Supplement: Supplementary file 1 [file research.1398.f1.zip › Supplementary materials.docx]

**Supplementary Figures and Figure legend**

**Figure S1.** **Characterization of senescent PVECs from aged mice.**

A) Schematic diagram illustrating the isolation of mouse primary PVECs from aged (18-month-old) mice. B) Flow cytometry assessment of purified PVECs. C-D) SA-β-gal staining was assessed in the mouse primary PVECs isolated from aged mice. (bar = 50 μm). E-F) Aging-related marker protein expression, including *p16*, *p19*, *p21*, *p53*, *IL-1β*, and *IL-18* mRNA in the PVECs, was detected by real-time PCR. G-H) Western blot for p53, p21, p16, and γ-H2AX protein expression in PVECs isolated from aged mice. *n* = 3. **P* < 0.05, ***P* < 0.01, and ****P* < 0.001.

**Figure S2.** **A metabolic shift from glycolysis to OXPHOS in D-gal-induced senescent endothelial cells.**

A) Flow cytometry assessment of purified PVECs following D-gal treatment. B-C) SA-β-gal staining was assessed in D-gal-treated mouse primary PVECs (bar = 20 μm). D) Senescence-related marker protein expression, including *p21*, *p53*, *Il-1β*, *Il-18,* and *Mmp2* mRNA in the mouse primary PVECs, was detected by real-time PCR. E-F) Western blot for p53, p21, p16, and γ-H2AX protein expression. *n* = 6. G) Analysis of lactate production in D-gal-treated mouse primary PVECs. H-I) OXPHOS-related proteins: ATP5A, UQCRC2, MTCO1, SDHB, and NDUFB8 in D-gal-treated mouse primary PVECs were detected by Western blot. *n* = 3. J) Schematic diagram of D-gal-treated HUVECs. K-L) SA-β-gal staining was assessed in D-gal-treated HUVECs (bar = 20 μm). M-P) Western blot for senescence-related proteins and OXPHOS-related proteins in D-gal-treated HUVECs. *n* = 3. **P* < 0.05, ***P* < 0.01, and ****P* < 0.001.

**Figure S3.** **Enhanced PDHA1 activity in D-gal-induced senescent endothelial cells.**

A) Relative PDHC activity in D-gal-treated mouse primary PVECs. B-C) The phosphorylation level at PDHA1^S293^ was determined in D-gal-treated mouse primary PVECs. *n* = 3. D) Relative PDHC activity in D-gal-treated HUVECs. E-F) The phosphorylation level at PDHA1^S293^ was determined in D-gal-treated HUVECs. *n* = 3. **P* < 0.05, ***P* < 0.01, and ****P* < 0.001.

**Figure S4.** **Generation of endothelial-specific conditional *Pdha1*-null mice.**

A) The endothelial-specific deletion of *Pdha1* was confirmed by PCR analysis of genomic DNA isolated from candidate mice. B) PDHC activity decreased in mouse primary PVECs isolated from *Pdha1*^ΔEC^. C) Mice were given a daily subcutaneous injection of D-gal (150 mg/kg) in the neck for 6 weeks. IF was employed to detect PDHA1 expression in endothelial cells in *Pdha1*^ΔEC^ mice (bar = 50 μm). D-E) Detection of PDHA1 expression in alveolar epithelial cells and macrophages from *Pdha1*^WT^ and *Pdha1*^ΔEC^ mice (bar = 20 or 50 μm). *n* = 6 mice/group.

**Figure S5. Endothelial‑specific deletion of *Pdha1* alleviates D‑gal‑induced lung histopathological changes.**

A) Representative images of H&E‑stained lung sections from *Pdha1*^WT^ and *Pdha1*^ΔEC^ mice after D‑gal treatment (bar = 100 μm). B–C) Quantification of lung injury scores. The destruction index (DI) and mean linear intercept (MLI) were used for quantitative analysis of pathological morphology (*n* = 6 mice).

**Figure S6.** **Inhibition of PDHA1 attenuates D-gal-induced cellular senescence *in vitro*.**

PVECs were exposed to PDHA1 competitive inhibitor 3-FP (15 μM) 1 h before D-gal (30 mg/mL) administration. A-B) SA-β-gal staining was assessed in the PVECs treated with 3-FP (bar = 20 μm). C-D) Western blot for p53, p21, p16, and γ-H2AX protein expression in 3-FP-treated PVECs. *n* =3. **P* < 0.05, ***P* < 0.01, and ****P* < 0.001.

**Figure S7.** **AZD7545 synergizes with D‑gal to promote senescence in PVECs.**

A) Representative Western blots showing the protein levels of p53, p21, p16, and γ‑H2AX in PVECs treated with increasing concentrations of D‑gal (0, 15, 30, 60 mg/mL). α‑Tubulin served as the loading control. B) Quantification of p53, p21, p16, and γ‑H2AX protein levels from (A). C) Representative Western blots showing the protein levels of the indicated proteins in PVECs treated with increasing concentrations of AZD7545 (0, 10, 20, 40 μM). D) Quantification of p53, p21, p16, and γ‑H2AX protein levels from (C). E) Representative Western blots showing the protein levels of the indicated proteins in PVECs treated with vehicle, D‑gal (15 mg/mL), AZD7545 (20 μM), or their combination. F) Quantification of p53, p21, p16, and γ‑H2AX protein levels from (A). *n* =3. **P* < 0.05, ***P* < 0.01, and ****P* < 0.001.

**Figure S8.** **Oligomycin reverses AZD7545‑induced upregulation of senescence markers.**

A) Representative Western blot images showing the protein levels of p53, p21, p16, and γ‑H2AX in PVECs treated with AZD7545 in the presence or absence of oligomycin (an ATP synthase inhibitor, 1.5 μM). α‑Tubulin served as the loading Control. B-E) Quantification of p53, p21, p16, and γ‑H2AX protein levels from (A). *n* =3. ****P* < 0.001.

**Figure S9. The phosphodeficient PDHA1-S293A mutant** **induces senescence in PVECs.**

Mouse primary PVECs were transfected with PDHA1-S293A mutant. A) Flow cytometry assessment of purified PVECs with PDHA1-S293A mutant. B) PDHA1-S293A mutation increased PDHC activity in mouse primary PVECs. C-D) SA-β-gal staining was performed in PVECs transfected with PDHA1-S293A mutant, scale bar = 20 μm. E-F) The expression of p-PDHA1^Ser293^ and senescence-related proteins (p53, p21, p16, and γ-H2AX) was examined by western blot. α-tubulin was used as the internal control, *n* =3. **P* < 0.05, and ***P* < 0.01.

**Figure S10.** **PDK1 protein level is not significantly altered in PVECs from aged mice.**

A-B) The expression of PDK1 in PVECs from aged mice was detected by Western blot. C-D) The expression of PDK1 in D-gal-treated PVECs was detected by Western blot. α-tubulin was used as the loading control, *n*=3.

**Figure S11. Lactylation orchestrates PDHA1 hyperactivation in endothelial senescence.**

A) Western blot showing lactyl-lysine levels in mouse primary PVECs freshly isolated from aged (18-month-old) mice. B) Western blot showing lactyl-lysine levels in D-gal-treated mouse primary PVECs. C-D) Mouse primary PVECs were pretreated with lactate (10 mM) for 30 min, and then exposed to D-gal (30 mg/mL). Cell samples were harvested after 24 h. SA-β-gal staining was assessed in the D-gal-treated PVECs with vehicle or lactate (bar = 50 μm). E-F) Ki67 staining in D-gal-treated PVECs with vehicle or lactate (scale bar = 50 μm). G-H) Western blot for p53, p21, p16, and γ-H2AX protein expression in D-gal-treated PVECs in the presence or absence of lactate. I-J) PVECs were pretreated with lactate (10 mM) for 30 min, and then exposed to D-gal (30 mg/mL) for 24 h. The phosphorylation level at PDHA S293 was determined. K) Relative PDHC activity was assessed. *n* =3. **P* < 0.05, ***P* < 0.01, and ****P* < 0.001.

**Figure S12.** **PDHA1-K336R overexpression exacerbates D-gal-induced upregulation of senescence markers.**

A) Representative Western blot images showing the protein levels of p53, p21, p16, and γ-H2AX in D‑gal‑treated PVECs transfected with PDHA1‑K336R. α‑Tubulin served as the loading Control. B-E) Quantification of p53, p21, p16, and γ‑H2AX protein levels from (A). *n* =3. **P* < 0.05, ***P* < 0.01, and ****P* < 0.001.

**Figure S13.** **Inhibition of PDHA1 rescues D-gal-induced mitochondrial stress and suppresses cGAS-STING Activation.**

PVECs were pretreated with the PDHA1 competitive inhibitor 3-FP (15 μM) for 1 hour before D-gal (30 mg/mL) challenge. A) The mtROS in D-gal-treated PVECs were detected by a ROS kit, bar = 20 μm. B-C) Mitochondrial depolarization in endothelial cells as shown by JC-1 Staining, bar = 20 μm. D-E) Western blot and quantification for cGAS, STING, p-TBK1^Ser172^, TBK1, p-IRF3^Ser396^, and IRF3 protein expression. α-tubulin was used as the internal control. *n* =3. **P* < 0.05, ***P* < 0.01, and ****P* < 0.001.

**Figure S14.** **Corresponding quantitative analysis of SA‑β‑gal‑positive cells.**

A) D‑gal‑induced SA‑β‑gal activity in *Pdha1*^WT^ vs *Pdha1*^ΔEC^ PVECs. B) SA‑β‑gal activity in PVECs transfected with Ctrl shRNA or *Pdha1* shRNA, with or without D‑gal treatment. C) Effect of AZD7545 on basal SA‑β‑gal activity. D) SA‑β‑gal activity in PVECs overexpressing PDHA1-WT or the PDHA1-K336R mutant. E) SA‑β‑gal activity in *Cgas*^WT^ and *Cgas*^KO^ PVECs treated with or without AZD7545. *n* =3. **P* < 0.05, ***P* < 0.01, and ****P* < 0.001.
